# Unique patterns of trimethylation of histone H3 lysine 4 are prone to changes during aging in *Caenorhabditis elegans* somatic cells

**DOI:** 10.1371/journal.pgen.1007466

**Published:** 2018-06-18

**Authors:** Mintie Pu, Minghui Wang, Wenke Wang, Satheeja Santhi Velayudhan, Siu Sylvia Lee

**Affiliations:** 1 Department of Molecular Biology and Genetics, Cornell University, Ithaca, New York City, United States of America; 2 Computational Biology Service Unit, Cornell University, Ithaca, New York City, United States of America; Babraham Institute, UNITED KINGDOM

## Abstract

Tri-methylation on histone H3 lysine 4 (H3K4me3) is associated with active gene expression but its regulatory role in transcriptional activation is unclear. Here we used *Caenorhabditis elegans* to investigate the connection between H3K4me3 and gene expression regulation during aging. We uncovered around 30% of H3K4me3 enriched regions to show significant and reproducible changes with age. We further showed that these age-dynamic H3K4me3 regions largely mark gene-bodies and are acquired during adult stages. We found that these adult-specific age-dynamic H3K4me3 regions are correlated with gene expression changes with age. In contrast, H3K4me3 marking established during developmental stages remained largely stable with age, even when the H3K4me3 associated genes exhibited RNA expression changes during aging. Importantly, the genes associated with changes in H3K4me3 and RNA levels with age are enriched for functional groups commonly implicated in aging biology. Therefore, our findings suggested divergent roles of H3K4me3 in gene expression regulation during aging, with important implications on aging-dependent pathophysiologies.

## Author summary

Histone modifications, the specific chemical modifications on histone proteins, are key for regulating the packing of DNA, and thus have important influence on diverse biological processes. An intensely studied function of histone modifications is their contribution to regulating gene expression. Recent studies in diverse model organisms demonstrated that the global alterations of particular histone modifications, for instance H3K4me3, extend the lifespan of the organism. However, the underlying molecular mechanisms remain largely unclear. In this study, we monitored whether and how the genome-wide pattern of the histone modification H3K4me3 changes during aging in the somatic cells of the model organism *C*. *elegans*. We identified interesting and non-conventional patterns of H3K4me3, which span gene-bodies and are acquired during adulthood, that are particularly prone to changes with aging. This is contrasted to the well-studied H3K4me3 patterns that span transcriptional start sites and 5’ promoter regions and are established early during development, which remain stable with age. Consistent with the close association between H3K4me3 marking and active transcription, we observed that the age-dynamic H3K4me3 markings are highly correlated with corresponding RNA expression changes. Importantly, the genes that are associated with both H3K4me3 and RNA expression changes with age are over-represented for functional groups commonly implicated in aging biology. In summary, our findings revealed a lesser known pattern of H3K4me3 modification that can have important biological roles in aging.

## Introduction

Aging in diverse organisms is accompanied by alterations in gene expression profiles that correlate with age-dependent physiological changes [1–13]. A recent study in *C*. *elegans* showed that the precise regulation of gene expression plays an important role in longevity, and inhibition of aging-dependent gene expression drift extends lifespan [14]. Epigenetic mechanisms are key to gene expression regulation [15]. Previous studies in diverse models demonstrated that aging is accompanied by dynamic changes in DNA methylation, histone modifications, and small RNAs levels [16–22]. How epigenetic changes contribute to aging biology and longevity determination remain largely unknown.

Tri-methylation on histone H3 lysine 4 (H3K4me3) is a widely recognized active promoter mark [23]. H3K4me3 typically marks the 5’ end of genes, surrounding the transcriptional start sites (TSS)[23]. It was reported that over 80% of the genes marked with H3K4me3 at the promoter region are actively transcribed [24]. H3K4me3 has been implicated in regulating mRNA preinitiation complex formation and transcription activation [25], enhancer usage [26], and pre-mRNA splicing [27]. However, studies in yeast and mammalian cells showed that defects in major H3K4me3 histone modification enzymes do not result in changes in gene expression [28–30]. Therefore, whether and how H3K4me3 contributes to gene expression regulation remain unclear. Recent studies in mammals indicated that atypical, broad domains of H3K4me3 are likely important for maintaining cell identity [31] and the expression of specific genes [32,33].

In *C*. *elegans*, appropriate H3K4me3 marking is important for longevity. Inactivation of the major H3K4me3 methyltransferase complex results in extended lifespan, and in addition reduced expression of H3K4me3 demethylases shortens lifespan [34,35]. The longevity effect associated with altered H3K4me3 depends on a functional germline and is postulated to partly due to gene expression changes that impact fat metabolism [34,36,37]. The direct mechanisms whereby altered H3K4me3 markings result in lifespan changes remain unknown.

In this study, we investigated whether and how H3K4me3 change with aging in the somatic cells of *C*. *elegans*. We found that around 30% of the H3K4me3 enriched regions exhibit significant changes in H3K4me3 levels with age. The data showed that these age-dynamic H3K4me3 regions preferentially mark gene-bodies and are acquired during adulthood, a pattern that is distinct from the canonical promoter marking. When compared with parallel RNA-seq data, we found that the age-dependent changes of H3K4me3 are significantly correlated with gene expression dynamics during aging. In contrast, our data indicated that canonical H3K4me3 marking at promoter regions, which are generally established during early development, remain largely stable with age, even when the H3K4me3 associated genes exhibit age-dependent RNA expression changes. Gene ontology (GO) term analysis showed that the genes associated with both H3K4me3 and RNA expression changes during aging are enriched for functional groups commonly implicated in aging biology. Our findings suggest a possible mechanistic link between age-dependent H3K4me3 dynamics, gene expression changes, and physiological aberrations.

## Results

### Identification of age-dependent H3K4me3 changes in *C*. *elegans* somatic cells

We performed chromatin immunoprecipitation coupled with deep sequencing (ChIP-seq) to profile the genome-wide patterns of H3K4me3 in *C*. *elegans* somatic cells at the young and old age (S1 Table). We used whole worm extracts from the temperature-sensitive *glp-1(e2141ts)* mutant that produces very few germ cells at the non-permissive temperature [38] to avoid the interference from the germline. We examined three independent biological replicates at two time points: day 2 (D2) adults, a time when wild-type worms are highly reproductive, as the young stage point, and D12 adults, a time when ~10% of the population has started to die, as the old stage point. Pair-wise correlation analysis indicated that the biological replicates from each of the time points were highly consistent, with Pearson’s correlation coefficients between 0.83 to 0.93 (S1A Fig), indicating the high reproducibility of the experiments.

To compare the H3K4me3 profiles between young and old stages, we first performed a genome-wide correlation analysis. Normalized H3K4me3 levels were computed using H3 ChIP-seq data as a control. Pearson correlation analysis was performed using normalized H3K4me3 in 2 kb windows tiling across the genome. We found that the genome-wide pattern of H3K4me3 did not drastically change with age, as the overall correlation coefficients were all higher than 0.81 (S2A Fig). Nevertheless, the clustering analysis revealed that replicates from the same time point were clustered closer together (S2A Fig), suggesting noticeable time point differences. We additionally compared the overall correlation of the H3 and H3K4me3 ChIP-seq data between the two points (S1B Fig). The results indicated that the genome-wide pattern of H3, unlike that of H3K4me3, did not show age-dependent differences, and validated that the H3 ChIP-seq data can be effectively used to normalize the H3K4me3 data.

**Fig 1:**
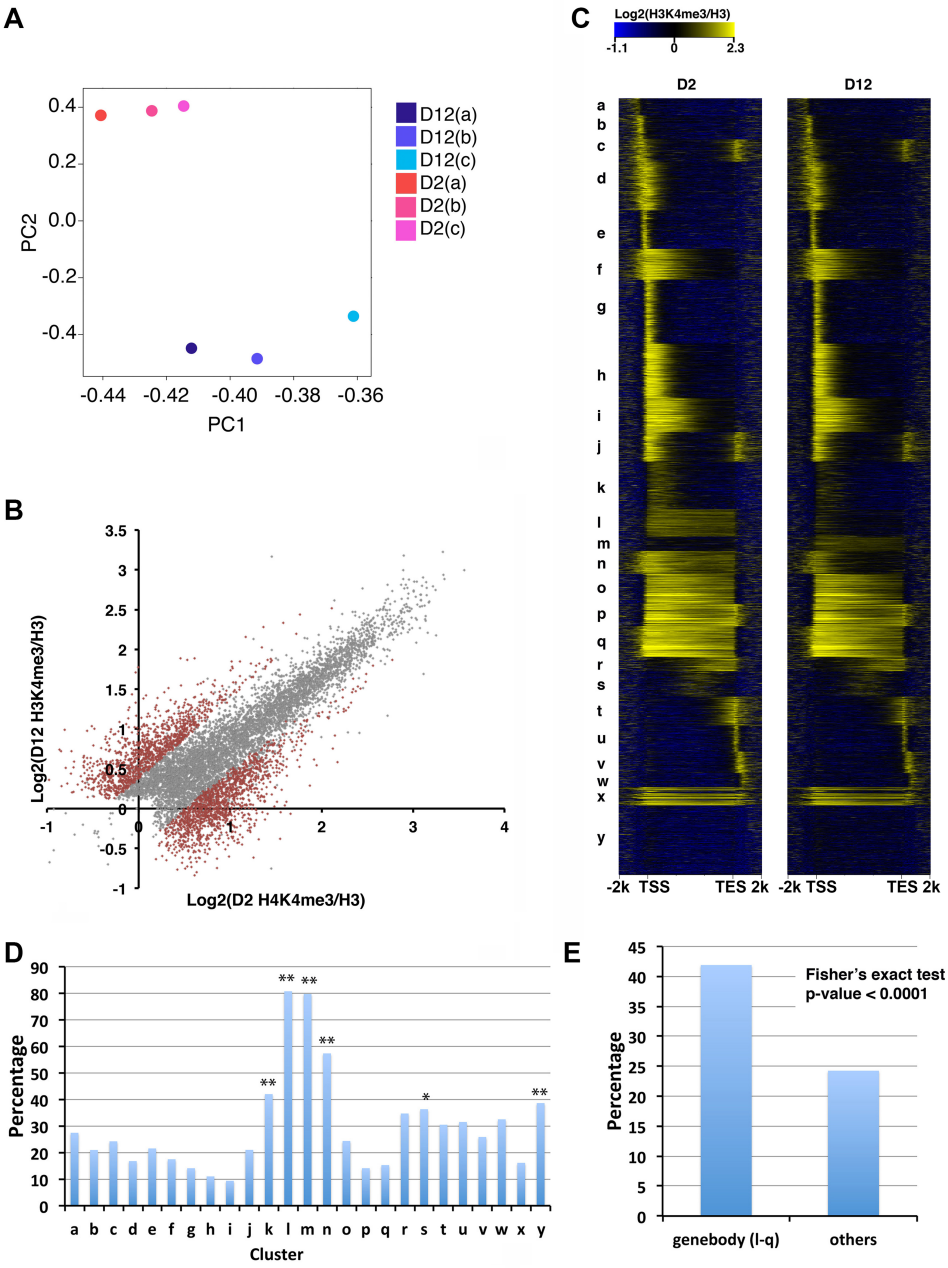
H3K4me3 markings on gene-bodies are enriched for age-dependent H3K4me3 changes. (A) PCA plot showing normalized H3K4me3 data from three biological replicates. The H3K4me3 peak regions at D2 and D12 were determined using the MACS2 narrow peak calling method. (B) Scatter plot showing normalized H3K4me3 levels at D2 and D12 for stable (grey) or dynamic (brown) H3K4me3 peaks. The plot shows the average normalized H3K4me3 signals from three biological replicates calculated using Homer. (C) Protein-coding genes associated with H3K4me3 peaks were grouped into 25 clusters (a-y) using k-means clustering based on their normalized H3K4me3 levels at D2 and D12 time points. The heatmaps show the log2 ratio of H3K4me3 levels normalized to H3 levels. (D) The bar chart shows the percentage of genes associated with age-dependent dynamic H3K4me3 peaks identified by DiffBind for each of the 25 clusters shown in (C). Clusters k, l, m, n, s and y are significantly enriched for genes associated with H3K4me3 peaks that dynamically changed with age. (*) p-value<0.05, (**) p-value<0.01, Fisher’s exact test. (E.) H3K4me3 peaks spanning gene-bodies are enriched for dynamic changes with age. The percentage of genes associated with age-dependent H3K4me3 change for clusters l to q, where H3K4me3 markings spanned gene-bodies, were compared with that for all the other clusters using Fisher’s exact test.

We next used MACS2 (2.1.0) to identify H3K4me3 enriched regions at each time point (S2 Table). Two different peak calling parameters were used to maximize the possibility of accurately defining the H3K4me3 enriched regions, which yielded the “narrow” and “broad” H3K4me3 peaks (S2 Table). In general, high concordance was observed for the sets of narrow and broad H3K4me3 peaks. Principal component analysis (PCA) was used to compare the H3K4me3 peaks identified from these 2 time points based on their covariance (Fig 1A and S2C Fig, narrow and broad peaks respectively). The PCA plots showed that the H3K4me3 enriched regions of D2 adults clearly separated from those of D12 adults along PC2 (Fig 1A and S2C Fig), supporting our earlier conclusion that the H3K4me3 markings at young and old age showed detectable differences.

To further identify the specific regions with significant age-dependent H3K4me3 changes, we used the DiffBind (v1.14.6)[39] statistical tool to perform the differential analysis. The narrow and broad peaks that showed significant time point differences were then merged, yielding 2,568 differential regions (S3 Table). Pearson correlation analysis of the normalized H3K4me3 signals within these differential regions confirmed that there were obvious differences between young and old stages (S2B Fig). Density plots of normalized H3K4me3 levels for the peaks that increased (1,168), decreased (1,400), or remained stable (5,152) with age showed that the H3K4me3 peaks that increased with age tended to be marked by low levels of H3K4me3 at D2, and the H3K4me3 peaks that decreased with age tended to be marked by medium levels of H3K4me3 at D2 (S2D Fig). The peaks marked with high levels of H3K4me3 at D2 tended to remain stable with age. Scatter plot and MA plots also demonstrated that the H3K4me3 peaks that showed significant age-dependent changes were marked by low to medium levels of H3K4me3 (Fig 1B and S2E Fig).

We next assigned the age dynamic H3K4me3 peaks to the closest annotated genes (S4 Table). The majority of the dynamic H3K4me3 peaks overlapped or spread into gene-body regions (2,190 peaks), whereas a small number of peaks (378) located in intergenic regions and were assigned to their closest downstream genes. 837 peaks overlapped with more than one gene and were assigned to more than one gene (S4 Table). The majority of the dynamic H3K4me3 peaks were assigned to protein-coding genes (S2F Fig).

### H3K4me3 peaks that show dynamic changes with aging are enriched for those that span gene-body

Since the majority of the dynamic H3K4me3 peaks were assigned to protein-coding genes (S2F Fig), we focused our downstream analyses using only the H3K4me3 peaks that were assigned to protein-coding genes. To identify any possible patterns associated with the age-dependent dynamic H3K4me3 peaks in an unbiased manner, we performed k-means clustering of the 9,213 protein-coding genes associated with H3K4me3 peaks (S4 Table). Normalized H3K4me3 levels (normalized to H3) spanning each gene together with 2kb upstream and downstream regions at the D2 and D12 time points were used for the clustering analysis. Empirical trials identified 25 clusters that represented the diversity of the H3K4me3 patterns for these genes (Fig 1C). The heatmaps revealed that, in addition to the typical H3K4me3 pattern that concentrated around annotated TSS (clusters a-j), there were also clusters with H3K4me3 signals spreading evenly across gene-body regions (clusters l-q) (Fig 1C). Similar clustering patterns were produced when H3K4me3 signals were normalized using input DNA (S3A Fig). We additionally presented the H3K4me3 distribution pattern in heatmaps oriented at the TSS, with gene order in the 25 clusters exactly the same as that in Fig 1C. The clustering results again supported that clusters l-q were represented by H3K4me3 markings spreading substantially downstream of TSS and into gene-bodies (S3C Fig).

The heatmaps showed that the H3K4me3 markings that spanned gene-body regions appeared enriched for dynamic changes with age, particularly clusters l, m, and n (Fig 1C). To further investigate this observation, we located the genes associated with the age-dynamic H3K4me3 peaks identified by DiffBind previously (2,544 genes) into these 25 clusters (S4 Table) and calculated the percentage of genes in each cluster with age-dynamic H3K4me3. Consistent with the visual representation, clusters l, m, and n, which contained genes marked by H3K4me3 throughout their gene-bodies, were significantly enriched for age-dependent H3K4me3 changes (Fig 1D). Clusters k and s, which showed weak partial gene-body H3K4me3 markings, were also significantly enriched for age-dependent H3K4me3 changes (Fig 1D). Fisher’s exact test confirmed that, as a group, the genes with gene-body H3K4me3 markings (clusters l to q) were associated with a more dynamic age-dependent change of H3K4me3 compared with genes with other H3K4me3 marking patterns (clusters a-k, r-y) (Fig 1E). Furthermore, average plot analysis showed that the H3K4me levels in clusters l, m, and n, were relatively lower than those of the other gene-body clusters ((o-q) (S2G Fig). Taken together, the data suggested that H3K4me3 markings that span gene-body regions and are at relatively lower levels are particularly prone to changes with age.

Due to this unusual pattern of gene-body spanning H3K4me3, we examined the average gene length and average H3K4me3 peak length for each of the 25 clusters, to rule out the possibility that the gene-body spanning pattern simply reflected conventional 5’ marking of H3K4me3 spreading over particularly short genes. In general, we observed variable average gene length for the different clusters (S3B Fig), but clusters k, l, and n contained genes that were of average or longer length, although cluster m contained genes that were relatively short. In addition, the peak length analysis indicated that clusters m and n were among the ones with the longest H3K4me3 peaks (S3D and S3E Fig). Therefore, the gene-body spanning pattern of H3K4me3 appeared specific and was not due to a technical caveat of the analysis method.

### Age-dynamic H3K4me3 markings are mainly deposited in adult stage

**Fig 2:**
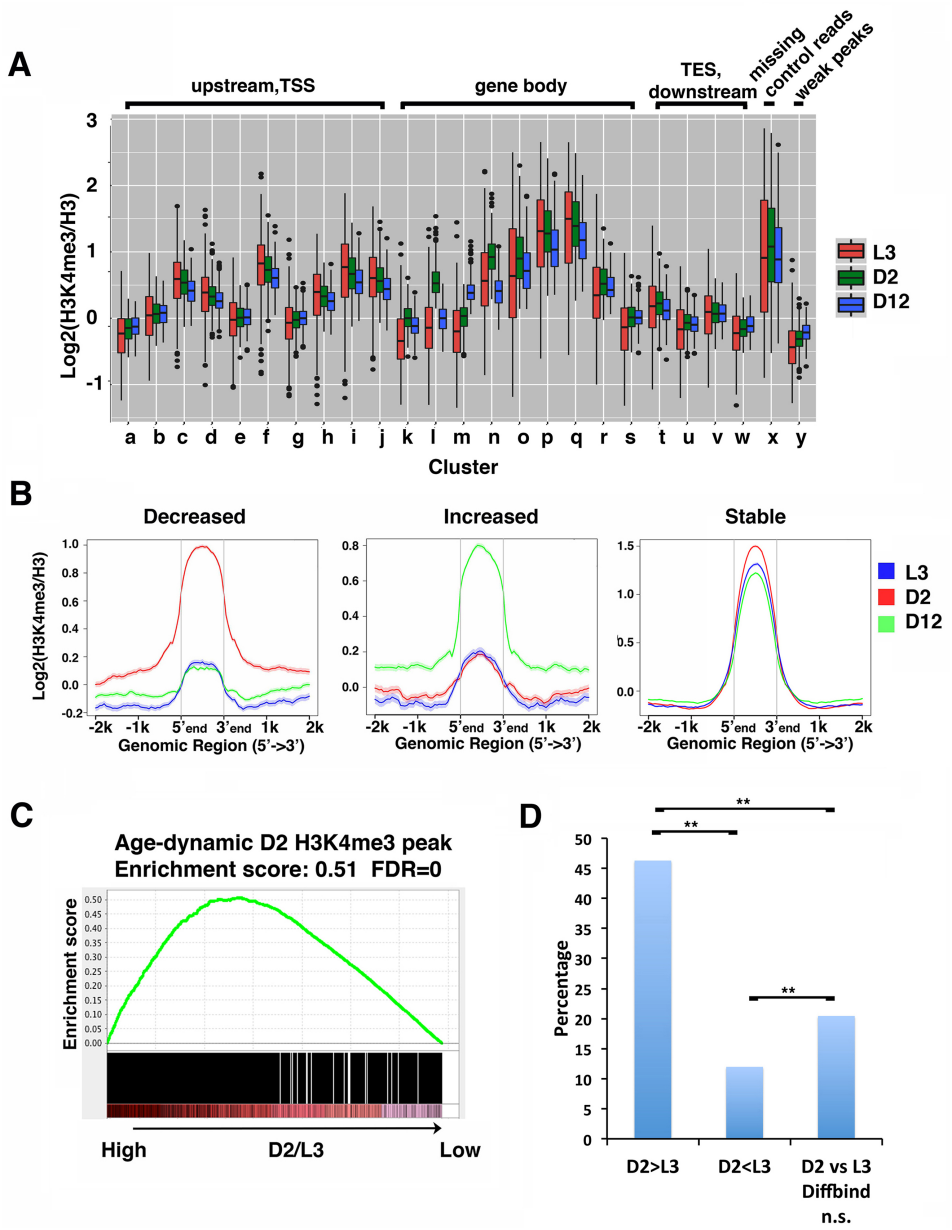
The age-dynamic H3K4me3 markings are mainly deposited in adults. (A) The boxplots show the normalized H3K4me3 levels in the 25 clusters at L3, D2 or D12 stages. (B) Average plots of the H3K4me3 levels at larval stage 3 (L3) (blue), D2 (red) and D12 (green) for stable peaks or peaks that showed significant dynamic changes with age identified by DiffBind. Normalized H3K4me3 levels within the peak regions and 2kb upstream and downstream are shown for the peaks that showed significantly decreased (left), increased (middle), or stable (right) H3K4me3 levels with age. (C) Age-dynamic H3K4me3 peaks are enriched for peaks marked with higher levels of H3K4me3 at D2 relative to L3. GSEA analysis was performed as described in Methods. The enrichment scores were computed using the GSEAPreranked tool. An enrichment score of 0.51 represents a statistically significant enrichment for peaks with a high D2/L3 H3K4me3 ratio. (D) H3K4me3 markings deposited during adult stage are enriched for age-dynamic H3K4me3 peaks. DiffBind was used to identify the H3K4me3 peaks with statistically significant differences between D2 and L3 stages. The differential (D2>L3, or D2<L3) or stable (D2 vs L3 ns) peaks were further compared with age-dynamic peaks to identify the overlapping peaks (S6 Table). The bar chart shows the percentage of age-dynamic H3K4me3 peaks for each of the groups. (**) p-value<0.0001, Fisher’s exact test.

To understand the possible temporal window that generates the different patterns of H3K4me3, we next compared the genome-wide H3K4me3 profiles in larval and adult stages in *C*. *elegans*. We performed H3K4me3 ChIP-seq in L3 stage *glp-1* mutant worms and identified 6,104 H3K4me3 peaks (S2 Table). We found that the majority of the H3K4me3 peaks detected in the L3 stage were maintained in adults. We further compared the H3K4me3 profiles at L3, D2 and D12 stages using the 25 clusters generated by k-means clustering discussed previously (S3A Fig) and computed the average normalized H3K4me3 levels for each cluster at the L3, D2, and D12 time points (Fig 2A). Strikingly, this comparison revealed that clusters k, l, m and n, which we found to be enriched for genes associated with age-dynamic H3K4me3 peaks (Fig 1), were marked with low or undetectable levels of H3K4me3 at the L3 stage compared to D2 or D12 (Fig 2A and S3A Fig), suggesting that the H3K4me3 markings for these gene clusters were mainly deposited during the adult stage.

To further examine the relationship between age-dynamic H3K4me3 regions with the timing of their initial deposition, we computed the average plots of the age-dynamic H3K4me3 peaks previously identified by DiffBind at the L3, D2 and D12 stages (Fig 2B). The data showed that for both the age-dependent increased or decreased groups, the H3K4me3 levels at L3 were significantly lower than that at D2 or D12 stages. In contrast, for the H3K4me3 peaks that remained stable with age, they were already marked with comparable levels of H3K4me3 at the L3 stage (Fig 2B).

We investigated this observation further using the statistical tool GSEA (Gene Set Enrichment Analysis) [40], which uses rank order to assess bias distribution, to test whether the age-dynamic H3K4me3 peaks were more likely to gain their H3K4me3 markings in the adult stage. The results supported that the age-dynamic H3K4me3 peaks were indeed significantly overrepresented for peaks with higher H3K4me3 markings at D2 compared to L3, indicating that the age-dynamic peaks were enriched for regions gaining H3K4me3 markings during adult stage (Fig 2C).

To analyze this observation in a converse manner, we examined whether the adult-stage deposited H3K4me3 regions could be enriched for age-dynamic changes. We used DiffBind to identify the H3K4me3 peaks with significant difference between D2 and L3 (S6 Table). We then compared the overlap between these L3/D2 differential peaks and the age-dynamic D12/D2 H3K4me3 peaks we identified earlier (S6 Table). The results showed that regions marked with significantly higher H3K4me3 levels at D2 stage relative to L3 were greatly enriched for age-dynamic peaks (Fig 2D). The peaks with higher H3K4me3 levels at L3 stage relative to D2 had the least overlap with the age-dynamic peaks (Fig 2D). Taken together, the data indicated that H3K4me3 deposited during developmental stages remain relatively stable during aging, whereas the H3K4me3 markings deposited in adult stage are more prone to change with age.

### Age-dependent H3K4me3 and gene expression changes are highly correlated

**Fig 3:**
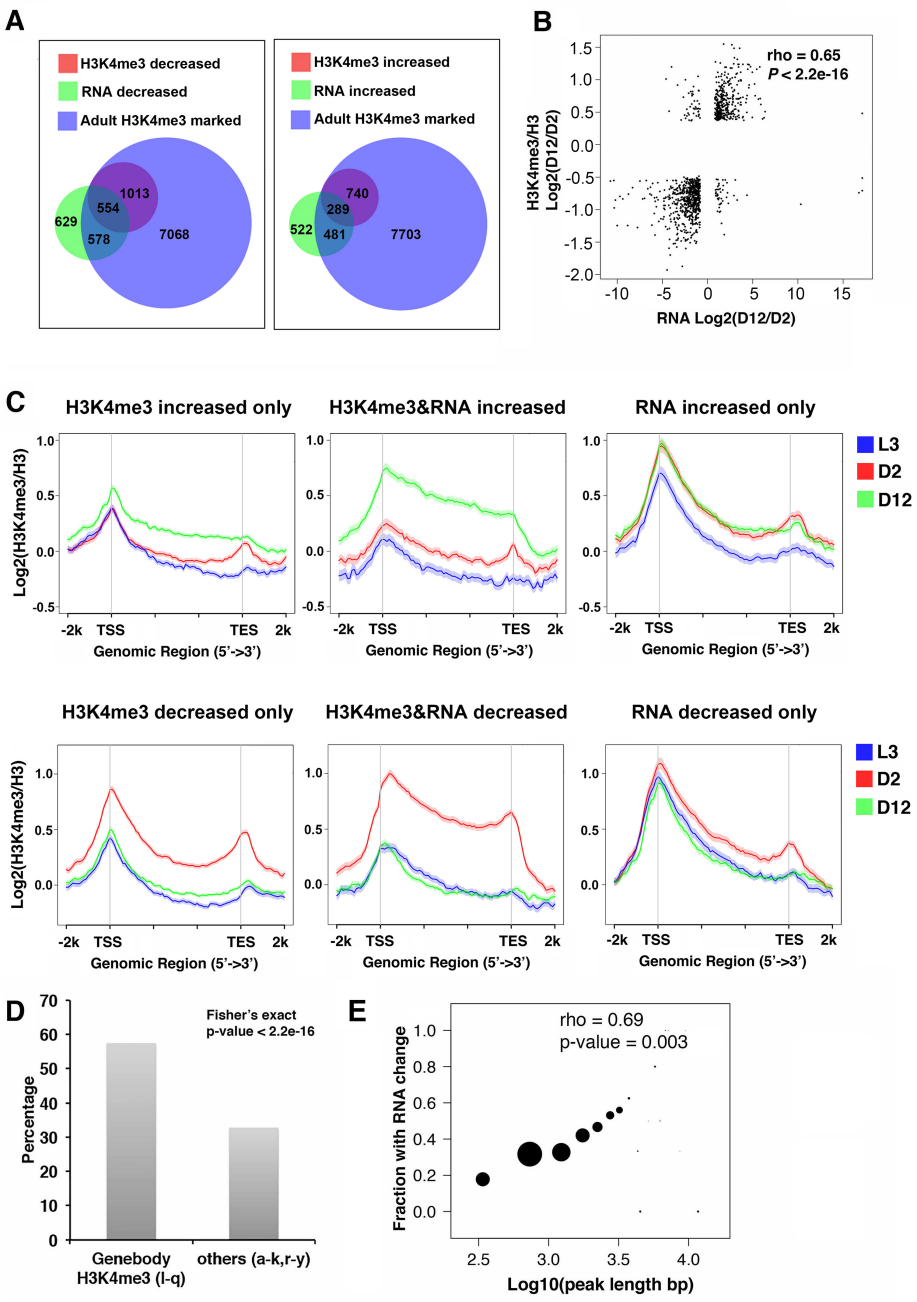
Age-dynamic H3K4me3 peaks accompanied by RNA expression change are more likely to span gene-body regions and be deposited during adult-stage. (A) The Venn diagrams show the protein-coding genes that were associated with H3K4me3 markings in adult stage (blue), significant H3K4me3 changes with age (red), and significant RNA expression changes with age (green). Gene numbers for each group are shown. (B) Age-dependent H3K4me3 changes and gene expression changes are positively correlated. Spearman’s correlation coefficient was calculated by using log2 ratio of normalized H3K4me3 levels and RNA expression levels at D12 relative to D2 for the age-dynamic peak regions and their assigned genes. (S8 Table). rho, Spearman’s correlation coefficient. (C) Genes associated with both age-dependent H3K4me3 and RNA expression changes tend to gain H3K4me3 markings in the adult stage. Average plots show normalized H3K4me3 levels at L3, D2, and D12 for gene groups associated with only age-dependent H3K4me3 changes (left), with both age-dependent H3K4me3 and RNA expression changes (middle), and with only age-dependent RNA expression changes (right). (D) The bar graph shows the percentages of age-dynamic H3K4me3 peaks that were accompanied by RNA expression changes in the l to q clusters (with gene-body H3K4me3) vs the other clusters. Fisher’s exact test shows that the dynamic H3K4me3 peaks in clusters l to q are significantly more likely to be accompanied by age-dependent RNA expression changes. (E.)The lengths of the age-dynamic H3K4m3 peaks are positively correlated with the likelihood of RNA expression change with age. Age-dynamic H3K4me3 peaks assigned only to one gene were used for this analysis. The y-axis represents the fraction of genes exhibiting age-dependent RNA expression change for each group of the indicated peak length (x-axis). The size of the dots indicates the number of peaks (also the number of genes) for each peak length range.

To investigate whether age-dynamic H3K4me3 changes correlated with gene expression changes, we performed parallel ribo-minus RNAseq analysis using the germlineless *glp-1* mutant worms at the exact time points used for the H3K4me3 ChIP-seq experiments (S7 Table). The overall correlations between the two biological replicates from each of the time points were both higher than 0.90, indicating the high reproducibility of the experiments. A comparison with our previously published poly-A RNAseq results with a similar experimental design [41] showed that the two RNAseq datasets were highly correlated at both the young and old time points (rho = 0.92 at D2 and D12) (S4A Fig). With the ribo-minus RNAseq data, the statistical tool edgeR identified 3,154 genes that showed significant expression change during aging. Among these, about half of the genes were marked with H3K4me3 (1,902), and among this subgroup, ~50% (919 genes) correlated with age-dependent H3K4me3 changes (S5A Fig). Importantly, the directions of RNA expression change and H3K4me3 change were significantly positively correlated, with a Spearman’s correlation coefficient of 0.65 (Fig 3B, S8 Table). Specifically, 554 genes exhibited corresponding decrease in RNA and H3K4me3 levels and 289 genes exhibited corresponding increase in RNA and H3K4me3 levels (Fig 3A). Only 86 genes showed opposing changes in RNA and H3K4me3 levels (S4B Fig).

Since H3K4me3 marking correlates well with active transcription[23], it was surprising that about half of the genes that showed age-dependent RNA expression change were not detectably marked by H3K4me3 in our data. To investigate this further, we compared publicly available ATAC-seq [42] and AMA-1 ChIP-seq data (*modENCODE2440*). ATAC-seq is commonly used to probe open chromatin regions [43] and AMA-1 is a subunit of RNA polymerase II (Pol II) in *C*. *elegans*. Both open chromatin and Pol II binding are usual features of active promoters, similar to H3K4me3 marking. In general, H3K4me3 markings in our data overlapped well with the ATAC-seq and AMA-1 ChIP signals (51.9% and 51.1% respectively). In contrast, for the genes that showed age-dependent RNA expression change but were not marked by H3K4me3, we observed significantly reduced ATAC-seq signals (28.6%) and AMA-1 occupancy (17%) (S5A Fig). These results indicated that the absence of H3K4me3 marking in these genes correlated with less open chromatin and reduced Pol II occupancy. We also noted that these genes exhibited lower average RNA abundance levels (S5B Fig).

### Adult-specific, gene-body H3K4me3 markings are enriched for age-dynamic H3K4me3 and gene expression changes

Although the age-dependent RNA expression changes and H3K4me3 changes were highly correlated, a substantial portion of the age-dynamic H3K4me3 peaks was associated with genes that showed no significant expression change with age based on our RNA-seq data (Fig 3A). Among the protein-coding genes associated with age-dynamic H3K4me3 peaks, 919 genes exhibited significant RNA expression change with age, whereas 1,625 showed stable expression (Fig 3A). To investigate the possible features that might distinguish these two groups, we first used average plots to examine the H3K4me3 levels of the age-dynamic H3K4me3 peak groups that were accompanied with or without RNA expression changes (Fig 3C). The data showed that, compared to the genes associated with only H3K4me3 dynamics, the genes associated with age-dependent changes in both H3K4me3 and RNA expression were marked with relatively higher levels of gene-body H3K4me3 coverage that were acquired during adult stage (Fig 3C and S4C Fig).

To test the statistical significance of this observable difference, we took advantage of the 25 clusters we previously discussed and tested whether the gene-body H3K4me3 clusters would be enriched for both dynamic H3K4me3 and gene expression changes with age. The results showed that, among the genes with dynamic H3K4me3, those in clusters l-q, where H3K4me3 obviously marked gene-body regions, were significantly enriched for genes with age-dependent RNA expression change compared with those in the other clusters (a-k, r-y)(Fig 3D). Furthermore, when individual clusters were evaluated, clusters k, l, m, and n, which were enriched for age-dynamic H3K4me3 peaks that cover gene-body (Fig 1D), contained significantly higher percentage of genes that showed both H3K4me3 and RNA expression change (S4D Fig). Interestingly, cluster p, which contained genes marked by high levels H3K4me3 through gene-body, while not enriched for age-dynamic H3K4me3 peaks (Fig 1C), nevertheless contained a significantly higher fraction of genes that exhibited age-dependent changes in both H3K4me3 and RNA expression (S4D Fig). We further detected a positive correlation between the length of the age-dynamic H3K4me3 peaks and the fraction of those peaks associated with genes expression change with age (Fig 3E). Taken together, the results suggested that broader regions of H3K4me3 marking, which tend to cover a greater portion of the gene-body and are mainly deposited in the adult stage, are more prone to dynamic changes with age and are also more likely to be accompanied by corresponding RNA expression changes.

### *ash-2* RNAi results in altered expression of genes with adult-stage specific H3K4me3

We next wondered whether the specific features of H3K4me3 that we discovered to correlate with age-dependent dynamic changes, including gene-body marking and adult-specific deposition, could have a role in regulating gene expression during adulthood. To test that, we re-analyzed previously published microarray data comparing the transcriptional profiles of the germlineless *glp-1(e2141ts)* mutant worms treated with or without *ash-2* RNAi at the D3 (day 3) adult stage [34]. ASH-2 is a component of the H3K4me3 methyltransferase complex in *C*. *elegans* and *ash-2* RNAi has been shown to cause a substantial global reduction of H3K4me3 [34]. Our analysis revealed that 831 and 981 protein-coding genes showed increased or decreased mRNA expression respectively upon *ash-2* RNAi (S9 Table). Since H3K4me3 marking is well known to associate with actively expressed genes, we posited that the upregulated mRNA expression following H3K4me3 depletion likely resulted from indirect effects, whereas the downregulated mRNA expression could reflect a more direct consequence of ASH-2 and H3K4me3 depletion.

**Fig 4:**
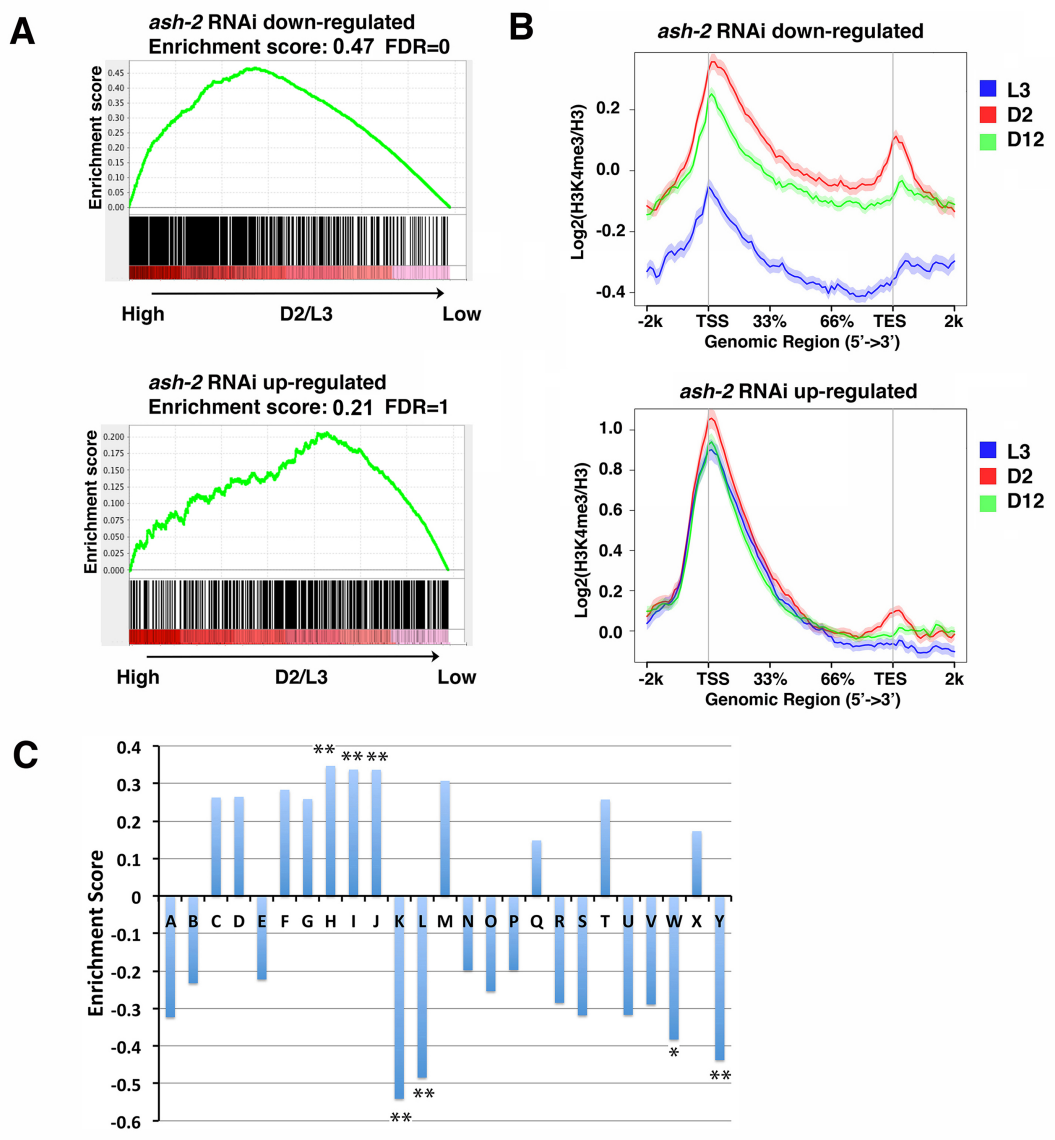
Genes that show downregulated expression when *ash-2* was knocked down are more likely to acquire H3K4me3 markings during the adult stage. (A) For genes exhibiting significantly downregulated expression when *ash-2* was knocked down, GSEA revealed a significant enrichment for genes associated with higher D2 H3K4me3 markings compared to L3 (top). For genes exhibiting significantly upregulated expression when *ash-2* was knocked down, GSEA revealed no bias (bottom). Genes with detectable expression from the published *ash-2* RNAi microarray experiment (GSM756690-GSM756695) were ranked according to the difference in H3K4me3 levels between D2 and L3 (S5 Table). The enrichment scores were computed using the GSEAPreranked tool. The genes that showed significant expression change (FDR <0.05) between *ash-2* and control RNAi were identified using Limma in the R package (S9 Table). (B) Average plots show that the genes that exhibited significantly downregulated expression upon *ash-2* RNAi (top) were marked with higher levels of H3K4me3 at D2 compared to L3. For the genes that exhibited significant upregulated expression upon *ash-2* RNAi (bottom), their average H3K4me3 levels did not change at the different developmental time points. (C) Clusters k, l, w and y were enriched for genes with a downregulated expression upon *ash-2* RNAi, and clusters h, i, and j were enriched for genes with an upregulated expression upon *ash-2* RNAi. Genes in all of the 25 clusters were ranked according to their expression fold change as detected in the *ash-2* microarray experiment. GSEA was used to determine the enrichment of downregulated or upregulated genes in each cluster. (**) FDR<0.01, (*) FDR<0.05.

We next intersected the candidate ASH-2-regulated genes with the gene lists we discussed earlier that associated with different features of H3K4me3 marking. Using GSEA, we found that the genes that exhibited downregulated mRNA expression when *ash-2* was knocked down were significantly enriched for genes marked by higher levels of H3K4me3 at D2 relative to L3, indicating that they were preferentially marked during adult stage (Fig 4A). Average plots of the normalized H3K4me3 levels at the L3, D2, and D12 stages for the genes that were downregulated when *ash-2* was knocked down also demonstrated a bias for H3K4me3 markings at the adult stage (Fig 4B). In contrast, the genes that exhibited upregulated expression when *ash-2* was knocked down did not show this bias (Fig 4A and 4B). Moreover, the ASH-2-mediated downregulated genes showed relatively low levels of H3K4me3 even during adulthood (Fig 4B, top), whereas the ASH-2-mediated upregulated genes were marked by high levels of H3K4me3 starting at L3 (Fig 4B, bottom). When we intersected the ASH-2 regulated genes with the 25 clusters previously defined (Fig 1), GSEA analysis showed that clusters k and l were significantly enriched for genes that became downregulated when *ash-2* was depleted (Fig 4C), and we showed earlier that clusters k and l were also enriched for genes that showed age-dynamic H3K4me3 markings that tended to be deposited in adults. The results together indicated that when *ash-2* was depleted through larval and adult stages, a small specific set of genes responded with expression change and the downregulated subgroup specifically correlated with adult-stage specific H3K4me3 deposition.

### Genes associated with age-dynamic H3K4me3 and RNA expression change are enriched for functional groups implicated in aging biology

To gain possible biological insights into the genes associated with age-dynamic H3K4me3 and RNA expression change, we performed gene ontology (GO) analysis using DAVID (S10 Table). Among the protein-coding genes associated with age-dependent H3K4me3 change, many functional clusters were significantly enriched for genes associated with decreased H3K4me3 with age, and fewer were associated with increased H3K4me3 with age (S10 Table). Similar functional clusters continued to be significantly overrepresented when considering the genes associated with both decreased H3K4me3 and RNA expression with age (S10 Table). Interestingly, many of these functional clusters have been implicated in aging biology, such as oxidation reduction, fatty acid metabolism, and mitochondrion. The most enriched functional clusters persisted when only genes with decreased gene-body H3K4me3 and RNA expression were considered (S10 Table).

**Fig 5:**
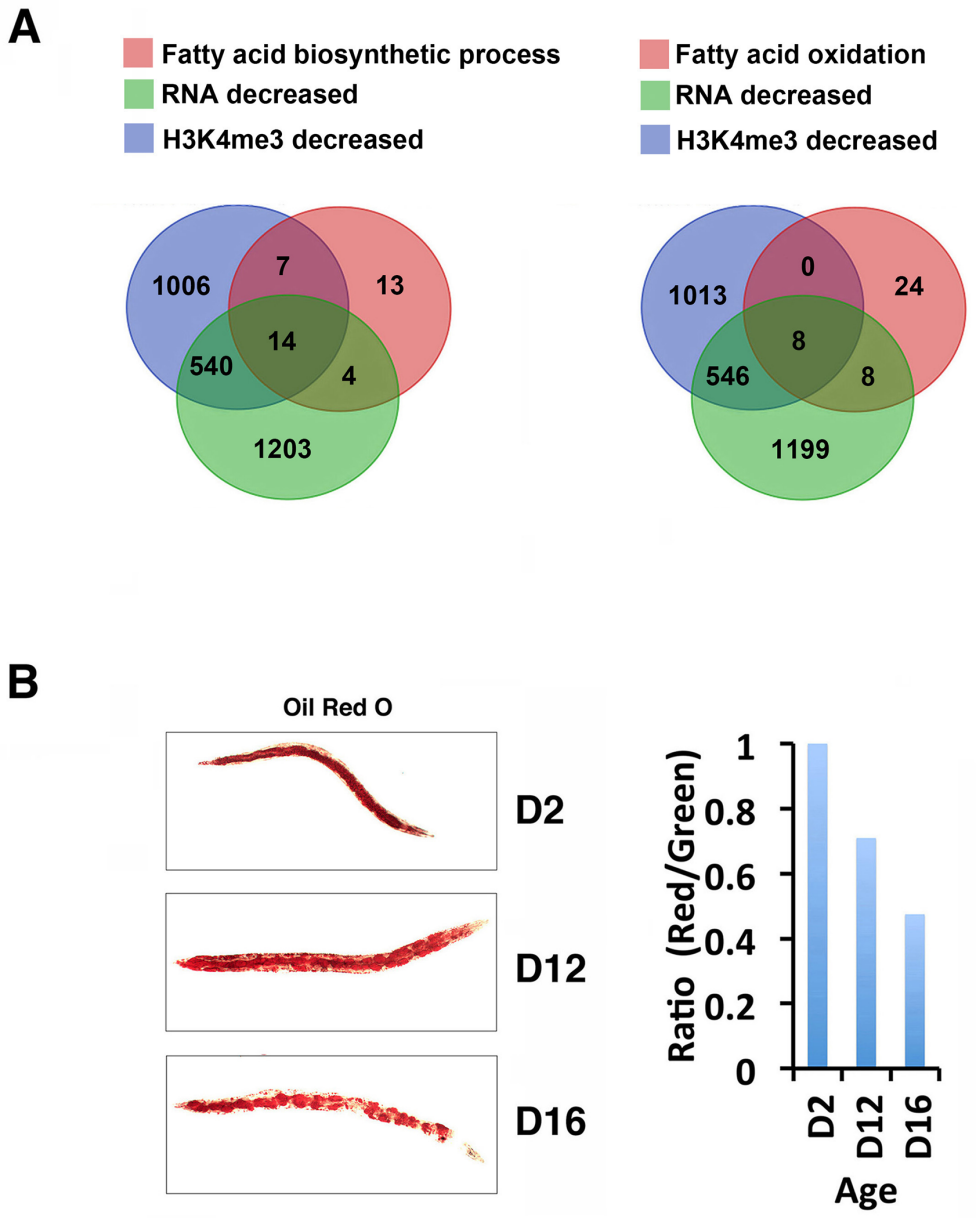
Fatty acid metabolism genes are overrepresented for age-dynamic H3K4me3 and gene expression. (A) A significant fraction of the genes annotated to act in the fatty acid biosynthetic (left) or fatty acid oxidation (right) pathways showed decreased RNA expression and/or H3K4me3 levels with age. Green: Genes with decreased RNA expression with age. Blue: Genes with decreased H3K4me3 levels with age. Red: Genes annotated to participate in fatty acid biosynthetic or fatty acid oxidation processes. Gene numbers of each group are shown in the Venn diagrams. (B) Oil-Red-O (ORO) staining of worms at D2, D12 and D16 aging time points (right). Quantification of the ORO signals (left) was done by normalizing the total signal counts in the red channel with the total signal counts in the green channel.

Since altered fat metabolism has been implicated in the role of germline H3K4me3 to modulate longevity [37,44,45], we further evaluated a possible connection between somatic H3K4me3 and fatty acid metabolism. A survey of WormBase identified 93 annotated fatty acid metabolism related genes that were marked by H3K4me3 in our data, and among them, ~49% showed age-dependent dynamic changes in H3K4me3 (46 out of 93 genes) (S11 Table). Furthermore, among the 46 genes, 25 were located in clusters l-n, with H3K4me3 mainly deposited on gene-bodies (S11 Table). We next identified the genes annotated to participate in fatty acid biosynthesis and fatty acid oxidation based on WormBase (S11 Table), and compared them with the age-dependent H3K4me3 and RNA expression upregulated or downregulated gene sets (Fig 5A). This comparison revealed that a significant fraction of the fatty acid biosynthesis and fatty acid beta-oxidation related genes showed age-dependent downregulation in H3K4me3 marking and RNA expression. Specifically, 37% of fatty acid biosynthesis related (14 genes out of 38 genes) and 20% of fatty acid oxidation related (8 genes out of 40 genes) genes exhibited reduced H3K4me3 and RNA abundance with age (S11 Table). In contrast, no enrichment for increased H3K4me3 and RNA expression was detected among these gene sets (S11 Table). The GO analyses suggested a possible deregulation of fat metabolism with aging. We monitored the fat content of *glp-1* mutants at various aging time points and confirmed that fat levels indeed decreased with age (Fig 5B).

## Discussion

In this study, we performed ChIP-seq analysis to identify dynamic changes of H3K4me3 with age in somatic cells of *C*. *elegans*. The data revealed that genome-wide H3K4me3 markings remain largely stable up until the D12 time point that we surveyed, but reproducible age-dependent changes in H3K4me3 markings are also readily detectable [46,47]. We found that ~30% of the H3K4me3 marked regions exhibit changes with age, and the dynamic changes generally happen in regions where H3K4me3 markings are relatively lower. In contrast, the regions marked by high levels of H3K4me3 remain largely stable with age. It is important to note that our experimental design, which used whole worm extracts, cannot distinguish regions marked by low levels of H3K4me3 in many cells vs. regions marked by high levels of H3K4me3 in a small subset of cells. Furthermore, our study took advantage of the germlineless mutant *glp-1(ts)*, which lacks notch signaling in the germline. In the future, it would be important to validate our findings in the somatic cells of wild-type worms, which would require either careful dissection of the soma or sorting of cells from specific tissues [48,49].

Our analyses revealed two major patterns of H3K4me3 marking: The canonical pattern where H3K4me3 marks concentrate around TSS with a bias towards the 5’ promoter region [23], and the atypical pattern where H3K4me3 marks span gene-bodies. For the gene-body spanning H3K4me3, we also observed either high or low levels of H3K4me3 markings. Our data indicated that the canonical pattern of H3K4me3, as well as the gene-body H3K4me3 with high levels of marking, are largely established by the L3 stage, and these H3K4me3 markings generally remain stable through the aging time points that we surveyed. In contrast, the weak or moderate levels of gene-body H3K4me3 markings are often acquired during adulthood and they exhibit dynamic changes with age. These adult-stage specific H3K4me3 marked regions could represent temporal-specific gene regulation and accompanied histone modifications. The low levels of H3K4me3 could reflect marking acquired in a specific subset of cells. How such temporal-/tissue-specific gene regulation then predispose genes for age-dependent changes will be an important avenue of future pursuit. Alternatively, these adult-specific, gene-body H3K4me3 regions could reflect cryptic transcription occurring in adult animals, which interfere with RNA expression in aged animals. Indeed, elevated levels of cryptic transcription have been shown to occur with advancing age in *C*.*elegans* [50]. Further studies to specifically probe the transcriptional status of genes at different aging time-points will help to distinguish these possibilities.

Histone modification marks are well known to act in concert to maintain local chromatin environment, we therefore compared the H3K4me3 profiles reported here with the H3K36me3 profiles we previously published [41]. As has been previously described, we identified regions co-occupied by both H3K4me3 and H3K36me3 around TSS and spanning gene-bodies [23]. This pattern of co-occupancy was already readily detectable at the L3 stage (S6A Fig). For the adult-specific H3K4me3 regions, which exhibit greater dynamics with age, we observed low or undetectable levels of H3K36me3 (S6A and S6B Fig). These genes are generally actively expressed in adults but are more likely to show expression changes with age (S6A Fig). This observation is exactly consistent with our previous finding that H3K36me3 marking is necessary for maintaining gene expression stability during aging and genes marked by low or undetectable levels of H3K36me3 show greater age-dependent RNA expression dynamics [41,50].

Since the age-dynamic H3K4me3 regions appear to be adult-stage specific, we also examined the genome-wide pattern of the histone variant H3.3 (HIS-72 in *C*. *elegans*) in somatic cells. Histone variant H3.3 is the major source for H3 turnover in post-mitotic cells and is thought to contribute to enhanced epigenetic plasticity and nucleosome dynamics [51]. We observed that the age-dynamic H3K4me3 regions usually have higher levels of H3.3 compared to the stable H3K4me3 regions (S6A and S6B Fig). In summary, we found that most of the H3K4me3 peaks that change with age are deposited in adult stages, and embedded in a chromatin environment with low levels of H3K36me3 and high levels of histone variant H3.3. These results pointed to possible temporal and spatial regulations of H3K4me3 deposition with important consequence on H3K4me3 stability with age. Further investigations into these possible mechanisms will help to better understand how coordinated histone modifications help to regulate chromatin environment dynamics during aging.

Our RNA-seq analysis revealed that ~7% of the protein-coding genes exhibit altered expression with age. Even though H3K4me3 is highly correlative with active transcription, we found that ~30% of the genes that showed significant expression change between D2 and D12 were not detectably marked by H3K4me3 at either time points (Fig 3 and S5A Fig). Additional analyses indicated that these genes were underrepresented for features commonly associated with active promoters, including open chromatin and Pol II occupancy (S5A Fig). We also noted that these genes exhibited lower average RNA expression levels (S5B Fig). Since all the analyses were done using whole worms, these genes could be lowly expressed or they could be expressed in a small number of cells. The absence / low levels of promoter features, including H3K4me3 marking, are consistent with the generally low RNA abundance levels of these genes.

It is important to note that ~70% of the genes that showed H3K4me3 changes were not accompanied by RNA changes. Conversely, ~50% of the genes that showed RNA changes were not accompanied by H3K4me3 changes. This observation is consistent with the current thinking that H3K4me3 marking is not instructive for gene expression, but rather represents a mark of transcriptional history. Following this thinking, changes in H3K4me3 would not be sufficient to induce RNA expression changes, and RNA expression changes do not necessarily have to be accompanied by H3K4me3 changes, depending on how quickly transcriptional “memory” follows transcriptional changes. Moreover, some of the changes detected in our RNA-seq data may not reflect transcriptional changes, but rather could be due to altered RNA processing and/or stability.

Despite that H3K4me3 is not thought to be instructive for gene transcription, the finding that a subset of genes show correlative changes in H3K4me3 and RNA expression levels with age led us to explore whether there could be a more direct regulatory relationship between H3K4me3 and RNA expression change. Using publicly available gene expression data associated with knockdown of the H3K4me3 methyltransferase *ash-2*, we identified an intriguing correlation between the genes that become downregulated upon *ash-2* RNAi and their unique pattern of H3K4me3 marking. We reasoned that since H3K4me3 is a histone mark that associates with active gene expression, if it had a regulatory role in promoting gene expression, its loss would be expected to result in decreased gene expression. Intriguingly, the subset of genes that decrease expression when H3K4me3 levels are reduced has a high tendency to be genes that normally acquire gene-body H3K4me3 marking during adulthood. While this finding cannot be used as definitive evidence of a cause-and-effect relationship between the H3K4me3 and RNA expression change with age, the data are certainly consistent with the model that altered H3K4me3 levels can have a regulatory role on the RNA expression of the specific subset of genes whose gene-body H3K4me3 marks are deposited during the adult stage. Studies in multiple organisms have shown that gene-body H3K4me3 is associated with RNA polymerase elongation [32,52,53]. It is possible that RNA polymerase elongation efficiency alters with aging, which could contribute to the age-dependent RNA expression changes observed for the subset of genes. Further investigation into this possibility has the potential to uncover a regulatory function of the major histone mark H3K4me3 in gene regulation, which thus far has remained enigmatic.

Although H3 lysine 4 methylation (H3K4me) is strongly associated with active transcription, recent studies in yeast indicate H3K4me represses gene expression. H3K4me2 and H3K4me3 have been suggested to collaborate to repress gene expression through promoting anti-sense transcription [28]. It is important to note that *ash-2* RNAi in *C*. *elegans* would also disrupt H3K4me2 deposition. Thus the gene activation we observed upon *ash-2* RNAi might be a consequence of losing H3K4me2 in addition to the absence of H3K4me3. Similar to H3K4me3, H3K4me2 is generally associated with active gene expression [23]. Compared to H3K4me3, H3K4me2 markings also typically locate around TSS, but extend further into the gene-bodies [23]. A Recent study revealed H3K4me2 and H3K4me3 markings in the gene-bodies of a subset of highly expressed muscle-related genes [54], suggesting a possible functional collaboration between gene-body H3K4me2 and H3K4me3. Future investigations of H3K4me2 profiles in the somatic cells of aging *C*. *elegans* will help to illuminate whether H3K4me2 and H3K4me3 cooperate during the aging process.

**Fig 6:**
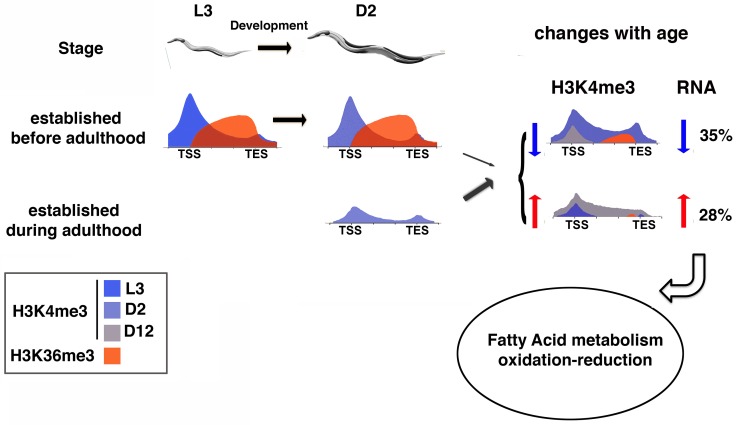
H3K4me3 markings established during adulthood are more likely to be age-dynamic and be accompanied by corresponding RNA expression change. Our data revealed that the H3K4me3 markings established before adulthood generally remain stable with age. These H3K4me3 marks exhibit the canonical pattern of accumulating to high levels around TSS and correlate with the presence of H3K36me3 markings in gene-bodies. In contrast, the H3K4me3 markings established in adult stage tend to be of lower levels and distribute more evenly into gene-bodies that are marked with very low or non-detectable levels of H3K36me3. The width of the gray arrow indicates the proportion of genes with significant age-dependent H3K4me3 change for each H3K4me3 marking pattern. For the genes associated with significantly increased (blue) or decreased (red) H3K4me3 levels with age, ~35% or 28% were accompanied by corresponding RNA expression change.

Our GO term analysis suggested that the genes that showed age-dynamic H3K4me3 and RNA expression change are enriched for functional groups commonly associated with aging biology, including fatty acid metabolism. Recent findings suggested that the elevated expression of the fatty acid desaturase enzymes FAT-5 and FAT-7 is essential for the lifespan extension caused by global inactivation of the H3K4me3 methyltransferase complex in wild-type reproductive worms [37]. Interestingly, when we inspected our data from the germlineless *glp-1* mutant worms, we noted that *fat-5* was marked with high levels of H3K4me3 on gene-body (cluster o) and its H3K4me3 marking and RNA expression remained stable with age. In addition, even though *fat-7* was actively expressed, it was not associated with detectable H3K4me3 marking. Therefore, our data indicated that *fat-5* and *fat-7* are not among the small subset of genes whose H3K4me3 and RNA expression show age-dynamic regulation. This finding is not surprising since the global reduction of H3K4me3 via inactivation of the methyltransferase is thought to act through the germline to regulate specific aspects of fat metabolism and to modulate lifespan. We speculate that in the somatic cells of *C*. *elegans*, a small subset of genes of particular biological importance, including fat metabolism, acquire adult-specific H3K4me3 markings that span gene-bodies and are dynamically regulated through aging. This age-dynamic H3K4me3 profile, in turn, regulates RNA expression changes, which likely contribute to specific physiological changes that accompany aging (Fig 6).

## Methods

### *C*. *elegans* strain growth and harvesting

*C*. *elegans* strain *glp-1(e2141)* was cultured at 16°C for stock propagation under standard growth conditions [55]. For ChIP-seq and RNA-seq experiments, embryos prepared from 16°C *glp-1(e2141)* worms by bleaching were hatched and cultured at 25°C with ∼3000 embryos per 15 cm of nematode growth medium (NGM) plates seeded with 1.5 mL of concentrated *E*. *coli* OP50 (30× overnight culture) with 50 μg/mL carbenicillin and 15 μg/mL tetracycline. For D12 samples, worms were refed once at D4 adult. Adult worms at the D2 and D12 stages were washed with ice-cold M9 three times. Worm pellets were frozen and stored at −80°C prior to ChIP and RNA extraction.

### ChIP and ChIP-seq library preparation

ChIP was performed as described [56,57]. Briefly, worm pellets were ground with a mortar and pestle and cross-linked with 1% formaldehyde in PBS for 10 mins at room temperature. Worm fragments were collected by spinning at 3,000g for 5 mins and resuspended in FA buffer followed by sonication with Bioruptor to generate chromatin fragments with major DNA length around 200 bp. Chromatin extracts were incubated with H3 (rabbit; Abcam, ab1791) or H3K4me3 antibodies (rabbit; Abcam ab9050) overnight at 4°C. Antibodies used were prescreened for specificity using dot blots. The optimal amounts of antibodies used were determined by titration in a preliminary experiment with ChIP-qPCR. Precipitated DNA (10–15 ng) from each sample was used for Illumina sequencing library preparation. DNA from ChIP was first end-repaired to generate a blunt end followed by adding single adenine base for adaptor ligation. The ligation products with adaptors were size-selected and amplified by PCR with primers targeting the adaptor. Up to 12 samples were multiplexed in one lane for single-end 50-nt Illumina HiSeq sequencing or single-end 75-nt Illumina NextSeq500. Raw ChIP-seq data have been deposited at Gene Expression Omnibus (GSE101964).

### ChIP-seq data analysis

ChIP-seq data quality control was performed (S1 Text and S7 Fig). Sequencing reads were filtered by FASTX_toolkit (-q 20 –p 80) and mapped to *C*. *elegans* reference genome (ce10) by bowtie2 (2.2.6) with the default setting [58] (bowtie2 -x ce10—U input_fastq—S output_sam). Mapped reads were extracted (samtools view -hS -F 4 input_same > output_sam) and PCR duplicates were removed with samtools (samtools rmdup -s input_bam output_bam) [59]. Aligned reads were further expressed as normalized bigwig using the deepTools [60] utility bamCompare with the parameters—binSize 25—fragmentLength 200—missingDataAsZero no—ratio subtract—scaleFactorsMethod readCount—normalizeUsingRPKM.

To compare the independent replicates of ChIP experiment, the mapped Illumina reads were counted in 2 kb sliding windows across the *C*. *elegans* genome in each experiment separately. The tag counts were normalized by the total number of aligned reads of each experiment, including the control experiments of H3, then multiply that by a million to get CPM. Lastly, log2 transformed CPM were calculated from treatment experiments and control experiments separately, and the control values were subtracted by corresponding treatments ones, which were used for calculating Pearson Correlation. Only the replicates with correlation values higher than 0.8 were used for further analyses. All statistical analyses were performed in R environment.

For H3K4me3 peak calling, mapped reads from biological replicates were merged for peak identification with MACS2 (2.1.0) [61]. Narrow and broad peaks were called by MACS2 with default settings for narrow peak calling or broad peak calling (for calling broad peaks: macs2 callpeak -t H3K4me3_bam -c H3_bam -n name_nomodle_duplication_auto—outdir—broad -g ce—broad-cutoff 0.1 -B—keep-dup auto -p 0.05 -m 2 50—nomodel—extsize 200; for calling narrow peaks: macs2 callpeak -t H3K4me3_bam -c H3_bam -n name_narrow_m550_nomode_—outdir -f BAM -g ce -B—call-summits—keep-dup auto -q 0.01 -m 5 50—nomodel—extsize 200). Peaks were then tested for consistency among three independent replicates using the GLM method in R package.

Verified peaks were used for differential analysis by DiffBind (v1.14.6) to identify the peaks that significantly changed between time points (dba.analyze(counts, method = DBA_EDGER_GLM, bFullLibrarySize = TRUE). The dynamic peaks that were identified from narrow peak calling or broad peak calling were merged if they overlapped by at least 1-bp. The dynamic peaks were further filtered using normalized histone modification levels and fold change between time points. To compute normalized histone modification levels, the H3K4me3 and H3 unique mapped reads were first normalized to total unique mapped reads in each library. The normalized read counts for individual peaks were calculated using the Homer software (annotatePeaks.pl peak ce10 -size given -d > output). The H3K4me3 read counts were further normalized with H3 read counts for each peak to yield the normalized H3K4me3 data (H3K4me3/H3). To compute fold change, Peaks with H3K4me3 and H3 ratio higher than 1.0 in all three replicates at either time points were kept. In addition, the peaks with normalized H3K4me3 levels increased or decreased by at least 30% during aging were kept for downstream analysis.

H3K4me3 peaks were assigned to the closest genes annotated in ce11 by Bedtools (bedtools closest -iu—a peak -b reference -D ref > output, bedtools closest -id—a peak -b reference -D ref > output). The genes assigned to H3K4me3 peaks located beyond their TES (transcription end site) were removed.

H3K4me3 profiles of protein-coding genes associated with H3K4me3 peaks at D2 and/or D12 adult were clustered into 25 clusters using ngsplot [62] (ngs.plot.r -G ce11 -R genebody/tss -C configure_file -L 2000 -D ensembl -SC global -GO km -KNC 25 –O output). The differences of normalized H3K4me3 levels of H3K4me3 peaks between D2 and L3 were ranked using ngsplot using the—GO diff setting.

### GSEA analysis

For GSEA analysis, GseaPreranked tool was used with the pre-ranked gene lists. For GSEA analysis in Fig 2, H3K4me3 peaks at D2 were ranked according to the ratio of H3K4me3 levels at D2 relative to L3 to generate the “ranked D2 H3K4me3 peaks” (S5 Table). Peaks with a higher ratio were assigned a higher rank. Age-dynamic H3K4me3 peaks (previously identified by DiffBind using D2 and D12 peaks, S3 Table) were then compared with the ranked D2 H3K4me3 peaks for overlapping. The ranked D2 H3K4me3 peaks that overlapped with the age-dynamic H3K4me3 peaks by at least 1 bp were used for enrichment score calculation.

For GSEA analysis in Fig 4A, genes with detectable expression from the published *ash-2* RNAi microarray experiment (GSM756690-GSM756695) were ranked according to the difference in H3K4me3 levels between D2 and L3 by ngsplot—GO diff setting. The ranked gene list was further used for GSEA analysis. For GSEA analysis in Fig 4C, the rank of RNA fold change in ash-2 RNAi was used for GSEA analysis (S5 Table).

### RNA-seq library preparation and data analysis

Total RNA was extracted from worms harvested at the same stages as ChIP-seq sample preparation for *glp-1(e2141)* worms using TRI reagent (Molecular Research Center). Total RNA was used for strand-specific library preparation with NuGEN kit (Ovation Human FFPE RNA-Seq Multiplex System). rRNAs were depleted using homemade oligos designed by NuGEN. 4 samples were multiplexed in one lane for single-end 100nt Illumina HiSeq sequencing. Raw RNA-seq data have been deposited at GEO (GSE101964).

For data analysis, tRNA and rRNA reads were first filtered out using Bowtie2 (bowtie2—un-gz output—al-gz rtRNA.mapped.fastq.gz -x rRNA&tRNA_reference -U input_fastq.gz—S rtRNA.mapped.sam). Libraries were stranded. RNA-seq reads were further aligned to WBcel235 transcript annotation by TopHat2 (v2.1.0) (—min-segment-intron 20—library-type fr-secondstrand—no-novel-juncs—transcriptome-index = transcriptome_data/known) with no novel junctions allowed [63]. The accepted aligned reads with a maximum of two mismatches were kept for differential expression analysis using edgeR. The raw read counts of each gene were input into edgeR for the analysis. (x <- read.delim(input)\group <- factor(c(1,1,2,2))\y <- DGEList(counts = x,group = group)\y <- calcNormFactors(y)\design <- model.matrix(~0+group, data = y$samples)\y <- estimateGLMCommonDisp(y,design)\y <- estimateGLMTrendedDisp(y,design)\y <- estimateGLMTagwiseDisp(y,design)\fit<-glmFit(y,design)\lrt<- glmLRT(fit, contrast = c(-1,1))\tab <- topTags(lrt, n = 46590)\write.table(output)).

### Gene ontology classification

Gene names were input into the Functional annotation-clustering tool in DAVID (6.8) (https://david.ncifcrf.gov/) [64] for gene annotation enrichment analysis. Functional annotation clustering was performed with the default criteria, and the enrichment score for each annotation cluster was determined. Functional annotation clusters with enrichment score higher than 1.3 and with GO term BH adjusted p-value <0.05 were reported.

### Oil-Red-O (ORO) staining of worms

Oil-Red-O staining was performed as described [65]. Worms were washed with M9 and stored at -80°C. For staining, worms were first fixed with 60% isopropanol and stained with freshly prepared Oil Red O working solution at 25 °C in a wet chamber for 6–18 hours. The Oil Red O working solution was removed and worms were then washed with 0.01% Triton X-100 in S buffer and kept in this solution at 4 °C before imaging.

## Supporting information

S1 Fig Related to Fig 1. (A)Pair-wise correlation plots showing the linear relationship between replicates. The H3K4me3 read counts were normalized by H3 read counts and library size. A 2kb sliding window size was used for calculating pair-wise Pearson correlation. The scatter plots show a pairwise pattern and the red lines represent the smooth regression lines (lower left panels). The correlation values are shown in the upper panels. (B) The consistency of H3 and H3K4me3 with age. (A) H3 reads or H3K4me3 reads were normalized to total mapped reads in each library and the normalized read counts were calculated in 2 kb sliding windows across the whole genome (A) or within peak regions showing significant changes with age (B). The pair-wise Pearson’s correlation coefficients were computed and shown in the heatmaps.
(TIF)

S2 Fig Related to Fig 1. (A) Genome-wide correlation analysis of H3K4me3 profiles in D2 (young) and D12 (old) germlineless *glp-1(ts)* worms. Pair-wise Pearson correlations of genome-wide H3K4me3 levels were calculated using 2kb sliding windows. (B)Correlation analysis of the peak regions that showed significant changes with age as identified by DiffBind. Normalized H3K4me3 levels were used for Pair-wise Pearson correlation analysis. (C)PCA plot showing normalized H3K4me3 data from three biological replicates. The H3K4me3 peaks in D2 and D12 were identified by the MACS2 broad peak calling method. (D)Density plots showing normalized H3K4me3 levels at D2 for peaks that showed increased (red), decreased (blue) or stable (black) modification levels with age. (E)The MA-plots depict the average H3K4me3 levels in log2-scale (x-axis) plotted against the difference between D2 and D12 in log2-scale (y-axis). The normalized H3K4me3 levels from narrow (left) or broad (right) peaks were used for the plots (MACS2 peak calling parameters described in Methods). The age-dynamic H3K4me3 peaks as determined by DiffBind EDGER-GLM analysis (FDR <0.05) are indicated as pink dots. The peaks that remained stable with age are presented as dark blue dots. (F)Each dynamic H3K4me3 peak was assigned to its closest gene as annotated in WBcel235. Assigned gene numbers of different gene types are shown. (G)Average plots show the normalized H3K4me3 levels for the indicated clusters. Clusters k, l, m, and n, which were enriched for age-dynamic H3K4me3 changes (shown in B), were marked with relatively lower levels of H3K4me3.
(TIF)

S3 Fig Related to Fig 2. (A)Heatmaps showing normalized H3K4me3 levels of 25 clusters of protein-coding genes by using input (left) or H3 (right) as the control at L3, D2 or D12. (B)Boxplots showing gene length in each cluster. (C)Heatmaps showing H3K4me3 distribution pattern centering around TSS with 5kb upstream and downstream (left) or proportional from TSS to TES (right, same as Fig 1C). The genes are ordered into 25 clusters exactly as that in Fig 1C. (D)Boxplots represent the peak length distribution of all age-dynamic peaks in each cluster. (E)Boxplots represent the peak length distribution of age-dynamic peaks uniquely assigned to each cluster.
(TIF)

S4 Fig Related to Fig 3. (A)Comparison of mRNA-seq data and ribo-minus RNAseq data from D2 or D12 *glp-1(e2141)* worms. The log10 (FPKM) values of mapped genes in both experiments were compared. (B)The Venn diagram shows the protein-coding genes that were associated with H3K4me3 marking in adult stages (blue), the ones associated with significant H3K4me3 change with age (red), and the ones associated with significant RNA expression change with age (green). Gene numbers for each group are shown. (C)Heatmaps showing normalized H3K4me3 signals at L3, D2 and D12 of the indicated gene groups with or without age-dependent H3K4me3 and/or RNA expression changes. The genes in each heatmap panel were ranked according to the ratio of H3K4me3 levels at D2 to that of L3. Peaks with higher ratios were placed at the top of the heatmaps. (D)The bar chart shows the percentage of genes associated with age-dynamic H3K4me3 that also exhibited age-dependent RNA expression change in each of the 25 clusters shown in Fig 1C. Clusters k, l, m, n, and p were significantly overrepresented for genes associated with age-dependent H3K4me3 and corresponding RNA expression changes.
(TIF)

S5 Fig Related to Fig 3. (A)Venn diagrams showing the overlap between the lists of protein-coding genes that showed age-dependent RNA expression change (green), marked by H3K4me3 in germlineless adult *glp-1* (blue), associated with age-dynamic H3K4me3 (red, left), ATAC peaks (red, middle) or AMA-1 occupancy (red, right). (B)Genes that showed age-dependent RNA expression changes but were not associated with H3K4me3 markings were generally expressed at lower levels. Boxplots show the RNA abundance distribution in each indicated gene group at D2 and D12.
(TIF)

S6 Fig Related to Fig 3. (A)Heatmaps showing normalized ChIP-seq signals of H3K4me3, H3K36me3, HIS-72::GFP and RNA abundance at the indicated stages in the 25 clusters described in Fig 1C. (B)Average plots show normalized H3K36me3, HIS-72::GFP and H3 signals within and surrounding H3K4me3 peaks that decreased (green), increased (red) or remained stable (black) with age.
(TIF)

S7 Fig Related to Fig 1. (A) Duplication plots for H3 ChIP-seq data. FASTQC module was used to analyze the duplication levels of each H3 ChIP-seq library and each plot shows the relative number of sequences with different degrees of duplication. First 100,000 sequences were analyzed for estimating the duplication levels in the whole data set. For each panel, the blue line is duplication distribution from all sequencing data, and the red one is from de-duplicated data. The proportion is the ratio of the deduplicated set relative to the original data. (B) Duplication plots for H3K4me3 ChIP-seq data. FASTQC module was used to analyze the duplication level of each H3K4me3 ChIP-seq library and each plot shows the relative number of sequences with different degrees of duplication. First 100,000 sequences were analyzed for estimating the duplication levels in the whole data set. For each panel, the blue line is duplication distribution from all sequencing data, and the red one is from de-duplicated data. The proportion is the ratio of the deduplicated set relative to the original data. (C)IDR analysis showing the consistency between replicates. H3K4me3 peaks were called by MACS2. Peaks from replicates at the same time point were compared and the results are shown for each pair. For each comparison: Upper Left: Replicates comparison based on peak ranks. The peaks that failed to pass the idr = 0.1 threshold are colored red. Upper right: Replicates comparison based on log10 peak scores. The peaks that failed to pass the idr = 0.1 threshold are colored red. Bottom left & right: Peaks rank versus idr scores are plotted from replicates. The boxplots display the distribution of idr values in each 5% quantile.
(TIF)

S1 Table Summary of ChIP-seq and RNA-seq data. (XLSX)

S2 Table MACS2 identified H3K4me3 peaks. (A) Adult narrow H3K4me3 peaks generated by merging D2 and D12 narrow MACS peaks with at least 1bp overlap. (B) Adult broad H3K4me3 peaks generated by merging D2 and D12 broad MACS peaks with at least 1bp overlap. (C) Adult H3K4me3 peaks by merging (A) and (B) with at least 1 bp overlap. (D) L3 H3K4me3 peaks by merging narrow and broad L3 MACS peaks with at least 1 bp overlap.
(XLSX)

S3 Table Age-dynamic H3K4me3 peaks identified by DiffBind. (A) Age-dynamic peaks identified by DiffBind with narrow D2 and D12 peaks. (B) Age-dynamic peaks identified by DiffBind with broad D2 and D12 peaks. (C) Age-dynamic peaks by merging (A) and (B) with at least 1 bp overlap.
(XLSX)

S4 Table Genes associated with H3K4me3 peaks. (A) Genes associated with adult H3K4me3 peaks. Merged adult H3K4me3 peaks in S2C Table were assigned to genes according to Methods. (B) Protein-coding genes from (A) with cluster assignments in Fig 1C. (C) Genes associated with age-dynamic H3K4me3 peaks. Age-dynamic peaks in S3C Table were assigned to genes according to Methods. (D) Protein-coding genes from (C) with cluster assignments in Fig 1C. (E) Genes in (D) with decreased H3K4me3 with age. (F) Genes in (D) with increased H3K4me3 with age.
(XLSX)

S5 Table Ranked lists for GSEA analysis in Figs 2C, 4A and 4C. (A) Ranked D2 H3K4me3 peaks for GSEA analysis in Fig 2C. ngsplot (-GO diff) was used to rank D2 H3K4me3 peaks according to their H3K4me3 difference between D2 and L3. The peaks with higher D2 H3K4me3 levels relative to L3 are assigned with higher ranks for GSEA. (B) Ranked gene list for GSEA analysis in Fig 4A. ngsplot (-GO diff) was used to rank genes according to their H3K4me3 difference between D2 and L3. The genes with higher D2 H3K4me3 levels relative to L3 are assigned with higher ranks for GSEA. (C) Ranked gene list for GSEA analysis in Fig 4C. Genes were ranked according to their fold change in RNA expression upon *ash-2* RNAi.
(XLSX)

S6 Table Overlapping between D2-L3 differential peaks and age-dynamic peaks. The overlapping between the peak subgroups below (A and B) and age-dynamic H3K4me3 peaks are listed in the table. (A) D2-L3 differential Peaks with H3K4me3 levels higher at D2 relative to L3 (B) D2-L3 differential Peaks with H3K4me3 levels higher at L3 relative to D2.
(XLSX)

S7 Table Genes that showed age-dependent RNA expression change. (A) Output of edgeR analysis of ribo-minus RNA-seq data (B) Differentially expressed genes (FDR<0.05) (C) Protein-coding gene with increased expression (D) Protein-coding gene with decreased expression.
(XLSX)

S8 Table Genes associated with age-dependent changes in both H3K4me3 and RNA expression used for scatter plot in Fig 3B. (XLSX)

S9 Table Genes that showed significant expression change upon *ash-2* RNAi. (XLSX)

S10 Table Functional annotations of genes that showed age-dependent H3K4me3 and RNA levels change. (XLSX)

S11 Table Lists of genes related to fatty acid metabolism in Fig 5. (A) GO terms and genes related to fatty acid metabolism (B) Protein-coding genes related to fatty acid metabolism from (A) marked by adult H3K4me3 (C) Protein-coding genes related to fatty acid metabolism from (B) with age-dynamic H3K4me3 (D) Genes related to fatty acid biosynthesis from (A) with/without H3K4me3 change and RNA expression change (E) Gene related to fatty acid oxidation from (A) with/without H3K4me3 change and RNA expression change.
(XLSX)

S1 Text Supplemental methods. (DOCX)
