# Supplementary figures and images for: Unique patterns of trimethylation of histone H3 lysine 4 are prone to changes during aging in Caenorhabditis elegans somatic cells
Source: PLoS Genet. 2018 Jun 18;14(6):e1007466. doi: 10.1371/journal.pgen.1007466 (PMC6023244; doi:10.1371/journal.pgen.1007466)

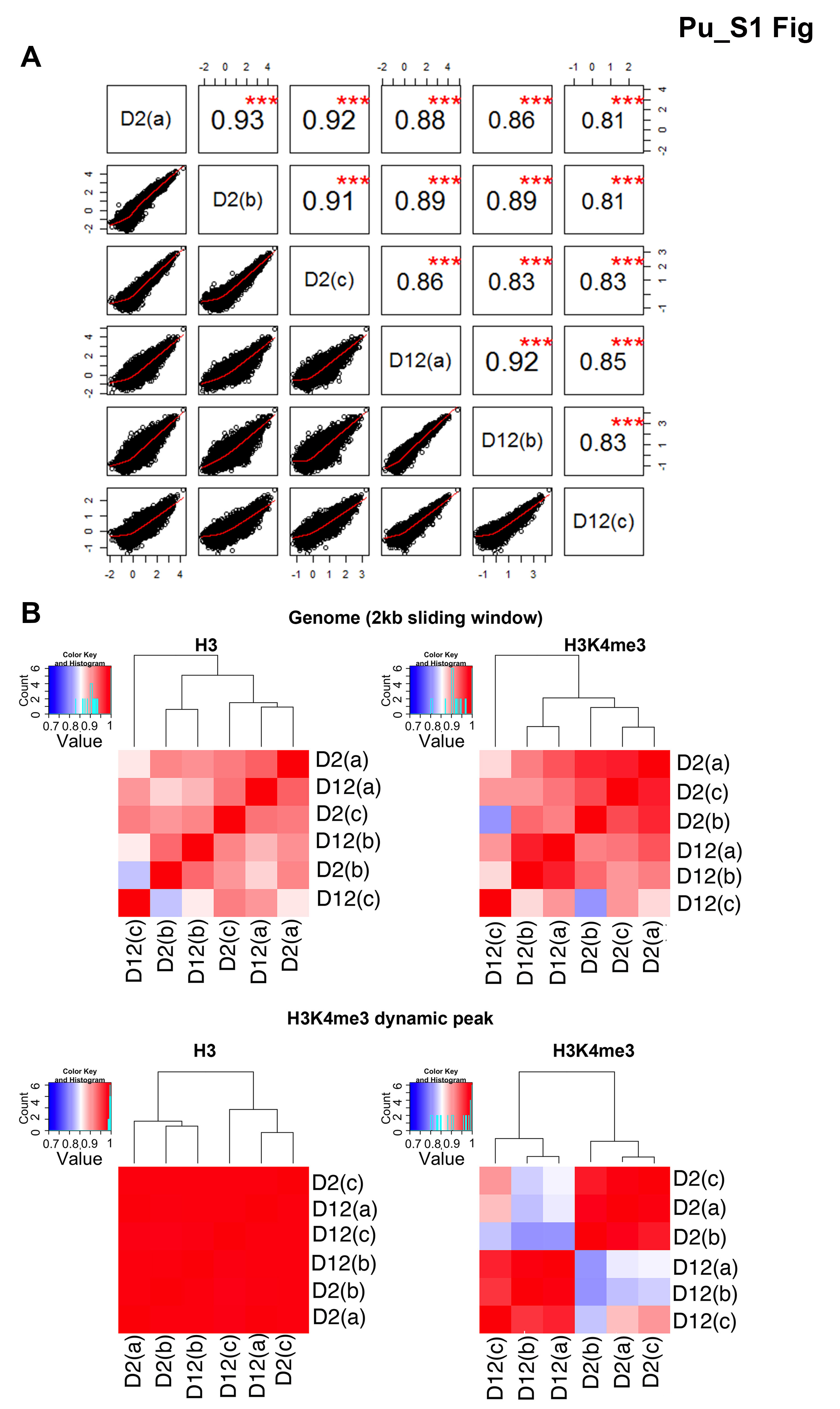

Supplement: S1 Fig — (A)Pair-wise correlation plots showing the linear relationship between replicates. The H3K4me3 read counts were normalized by H3 read counts and library size. A 2kb sliding window size was used for calculating pair-wise Pearson correlation. The scatter plots show a pairwise pattern and the red lines represent the smooth regression lines (lower left panels). The correlation values are shown in the upper panels. (B) The consistency of H3 and H3K4me3 with age. (A) H3 reads or H3K4me3 reads were normalized to total mapped reads in each library and the normalized read counts were calculated in 2 kb sliding windows across the whole genome (A) or within peak regions showing significant changes with age (B). The pair-wise Pearson’s correlation coefficients were computed and shown in the heatmaps. (TIF) [file pgen.1007466.s001.tif]

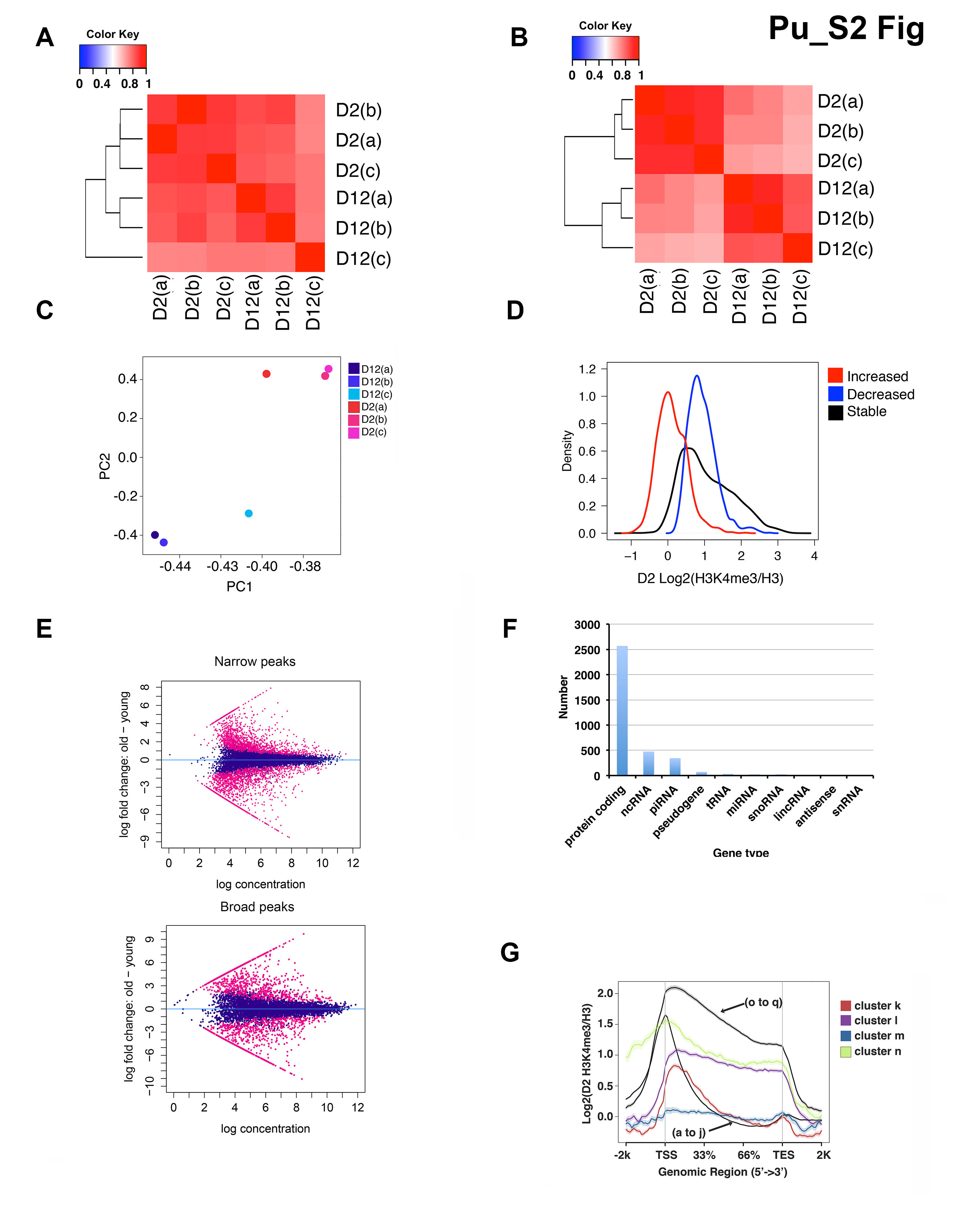

Supplement: S2 Fig — (A) Genome-wide correlation analysis of H3K4me3 profiles in D2 (young) and D12 (old) germlineless glp-1(ts) worms. Pair-wise Pearson correlations of genome-wide H3K4me3 levels were calculated using 2kb sliding windows. (B)Correlation analysis of the peak regions that showed significant changes with age as identified by DiffBind. Normalized H3K4me3 levels were used for Pair-wise Pearson correlation analysis. (C)PCA plot showing normalized H3K4me3 data from three biological replicates. The H3K4me3 peaks in D2 and D12 were identified by the MACS2 broad peak calling method. (D)Density plots showing normalized H3K4me3 levels at D2 for peaks that showed increased (red), decreased (blue) or stable (black) modification levels with age. (E)The MA-plots depict the average H3K4me3 levels in log2-scale (x-axis) plotted against the difference between D2 and D12 in log2-scale (y-axis). The normalized H3K4me3 levels from narrow (left) or broad (right) peaks were used for the plots (MACS2 peak calling parameters described in Methods). The age-dynamic H3K4me3 peaks as determined by DiffBind EDGER-GLM analysis (FDR <0.05) are indicated as pink dots. The peaks that remained stable with age are presented as dark blue dots. (F)Each dynamic H3K4me3 peak was assigned to its closest gene as annotated in WBcel235. Assigned gene numbers of different gene types are shown. (G)Average plots show the normalized H3K4me3 levels for the indicated clusters. Clusters k, l, m, and n, which were enriched for age-dynamic H3K4me3 changes (shown in B), were marked with relatively lower levels of H3K4me3. (TIF) [file pgen.1007466.s002.tif]

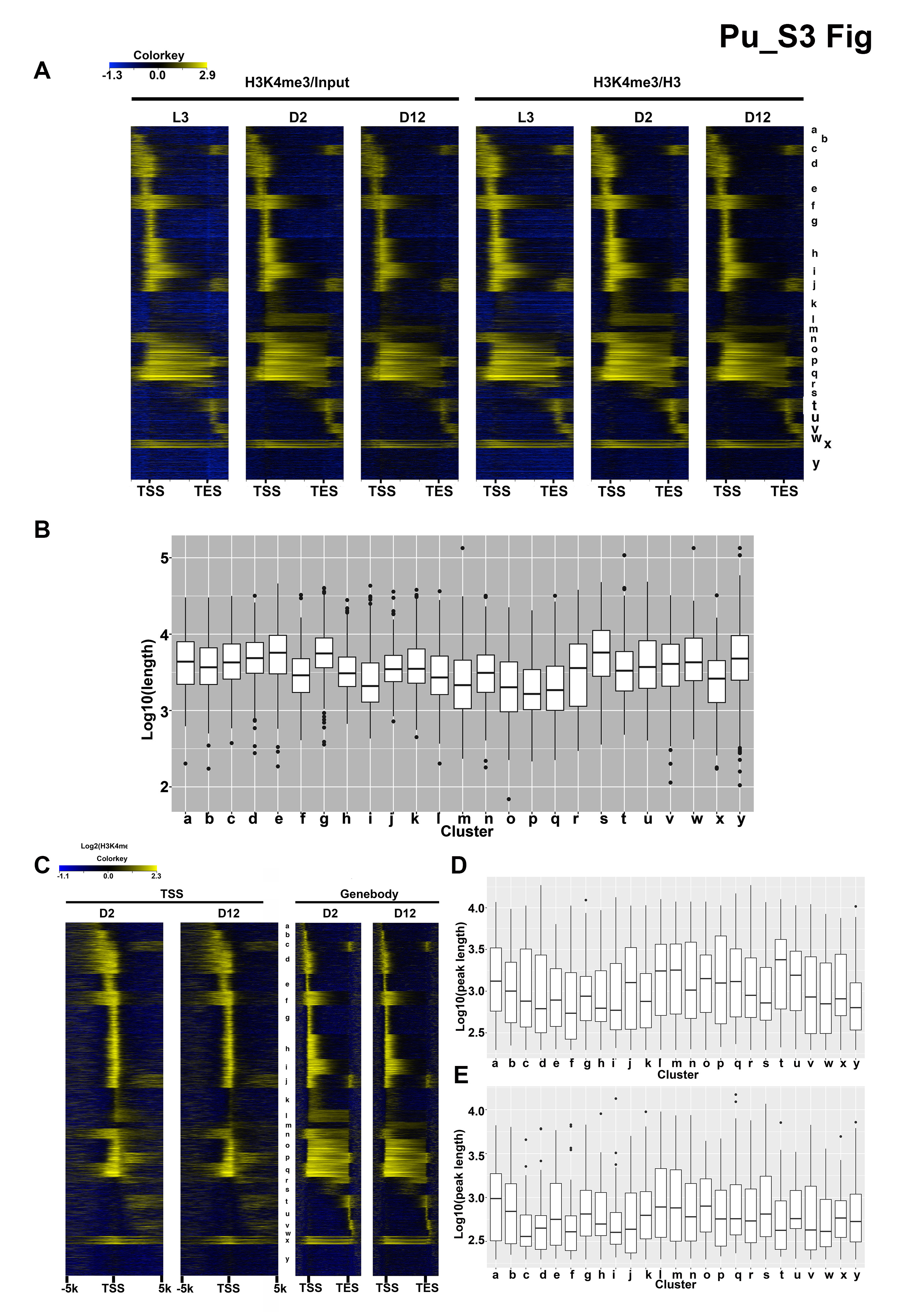

Supplement: S3 Fig — (A)Heatmaps showing normalized H3K4me3 levels of 25 clusters of protein-coding genes by using input (left) or H3 (right) as the control at L3, D2 or D12. (B)Boxplots showing gene length in each cluster. (C)Heatmaps showing H3K4me3 distribution pattern centering around TSS with 5kb upstream and downstream (left) or proportional from TSS to TES (right, same as Fig 1C). The genes are ordered into 25 clusters exactly as that in Fig 1C. (D)Boxplots represent the peak length distribution of all age-dynamic peaks in each cluster. (E)Boxplots represent the peak length distribution of age-dynamic peaks uniquely assigned to each cluster. (TIF) [file pgen.1007466.s003.tif]

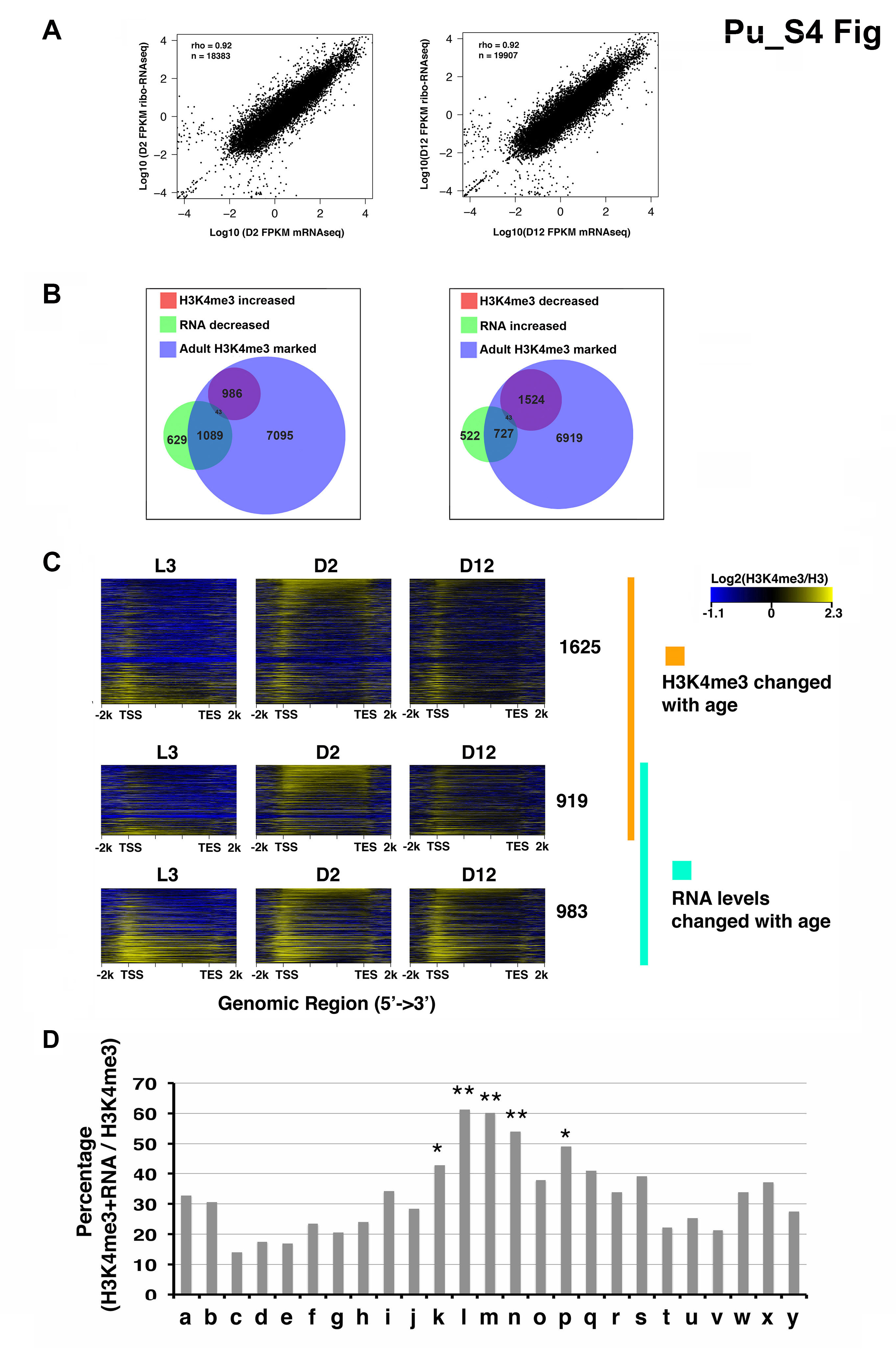

Supplement: S4 Fig — (A)Comparison of mRNA-seq data and ribo-minus RNAseq data from D2 or D12 glp-1(e2141) worms. The log10 (FPKM) values of mapped genes in both experiments were compared. (B)The Venn diagram shows the protein-coding genes that were associated with H3K4me3 marking in adult stages (blue), the ones associated with significant H3K4me3 change with age (red), and the ones associated with significant RNA expression change with age (green). Gene numbers for each group are shown. (C)Heatmaps showing normalized H3K4me3 signals at L3, D2 and D12 of the indicated gene groups with or without age-dependent H3K4me3 and/or RNA expression changes. The genes in each heatmap panel were ranked according to the ratio of H3K4me3 levels at D2 to that of L3. Peaks with higher ratios were placed at the top of the heatmaps. (D)The bar chart shows the percentage of genes associated with age-dynamic H3K4me3 that also exhibited age-dependent RNA expression change in each of the 25 clusters shown in Fig 1C. Clusters k, l, m, n, and p were significantly overrepresented for genes associated with age-dependent H3K4me3 and corresponding RNA expression changes. (TIF) [file pgen.1007466.s004.tif]

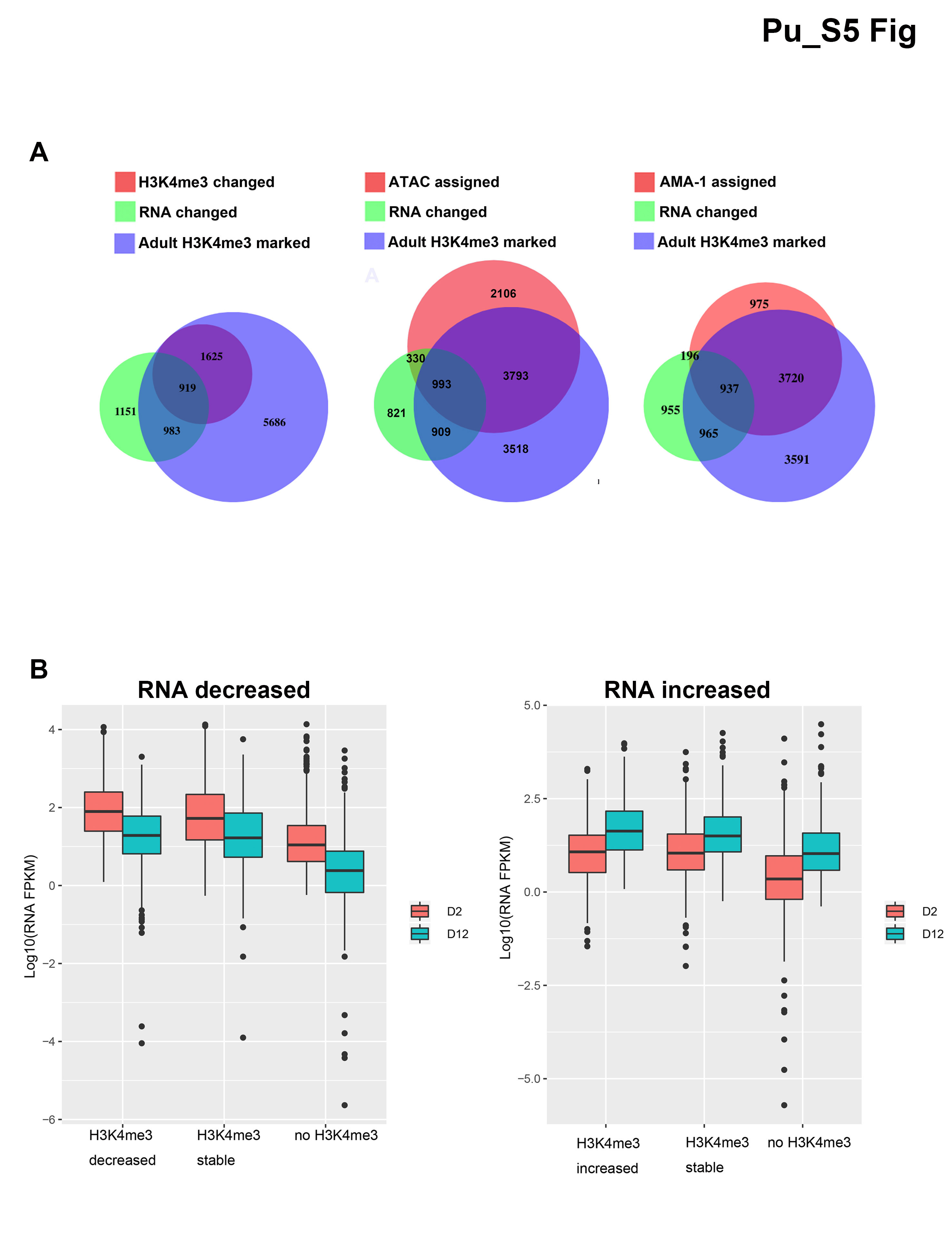

Supplement: S5 Fig — (A)Venn diagrams showing the overlap between the lists of protein-coding genes that showed age-dependent RNA expression change (green), marked by H3K4me3 in germlineless adult glp-1 (blue), associated with age-dynamic H3K4me3 (red, left), ATAC peaks (red, middle) or AMA-1 occupancy (red, right). (B)Genes that showed age-dependent RNA expression changes but were not associated with H3K4me3 markings were generally expressed at lower levels. Boxplots show the RNA abundance distribution in each indicated gene group at D2 and D12. (TIF) [file pgen.1007466.s005.tif]

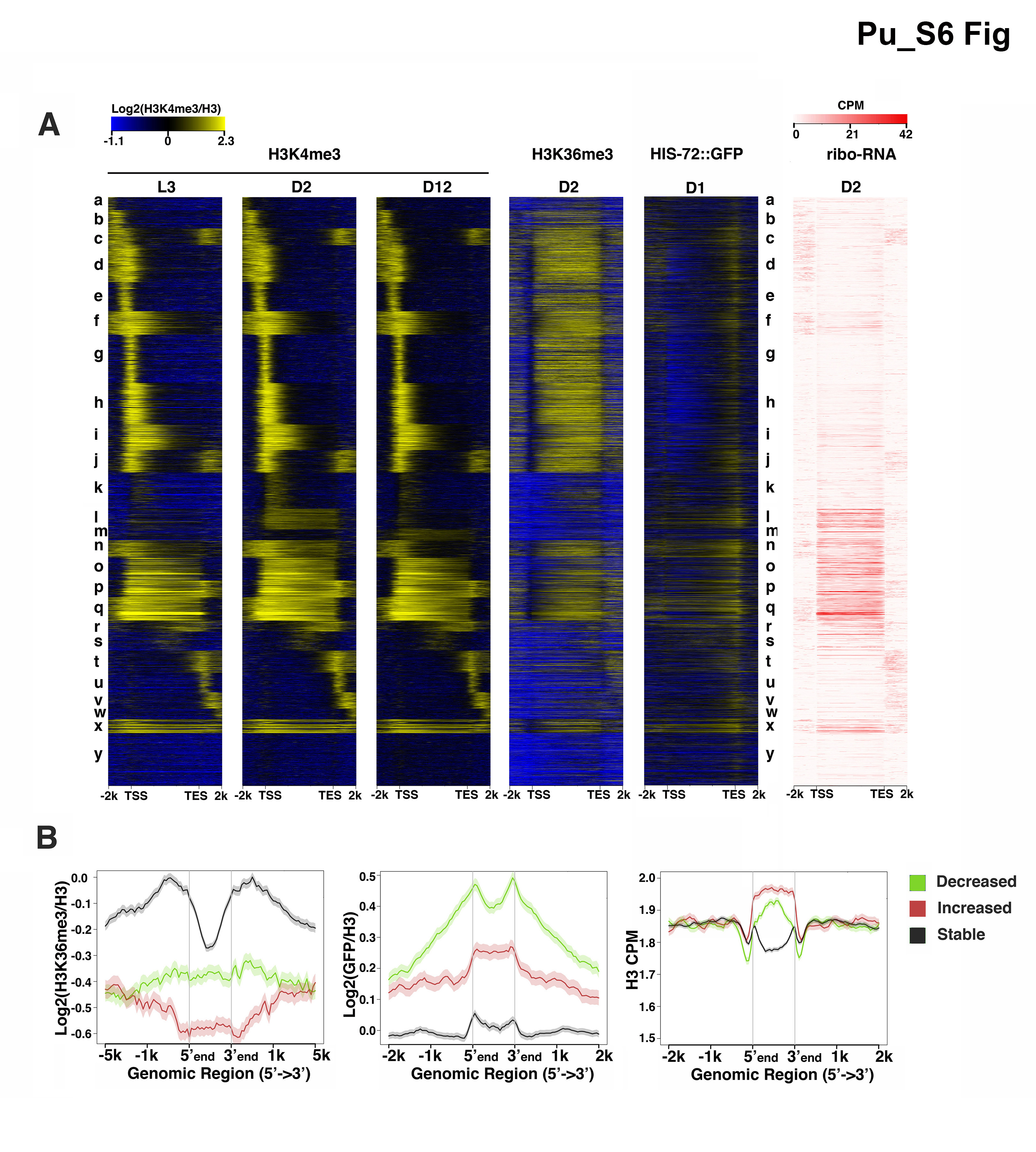

Supplement: S6 Fig — (A)Heatmaps showing normalized ChIP-seq signals of H3K4me3, H3K36me3, HIS-72::GFP and RNA abundance at the indicated stages in the 25 clusters described in Fig 1C. (B)Average plots show normalized H3K36me3, HIS-72::GFP and H3 signals within and surrounding H3K4me3 peaks that decreased (green), increased (red) or remained stable (black) with age. (TIF) [file pgen.1007466.s006.tif]

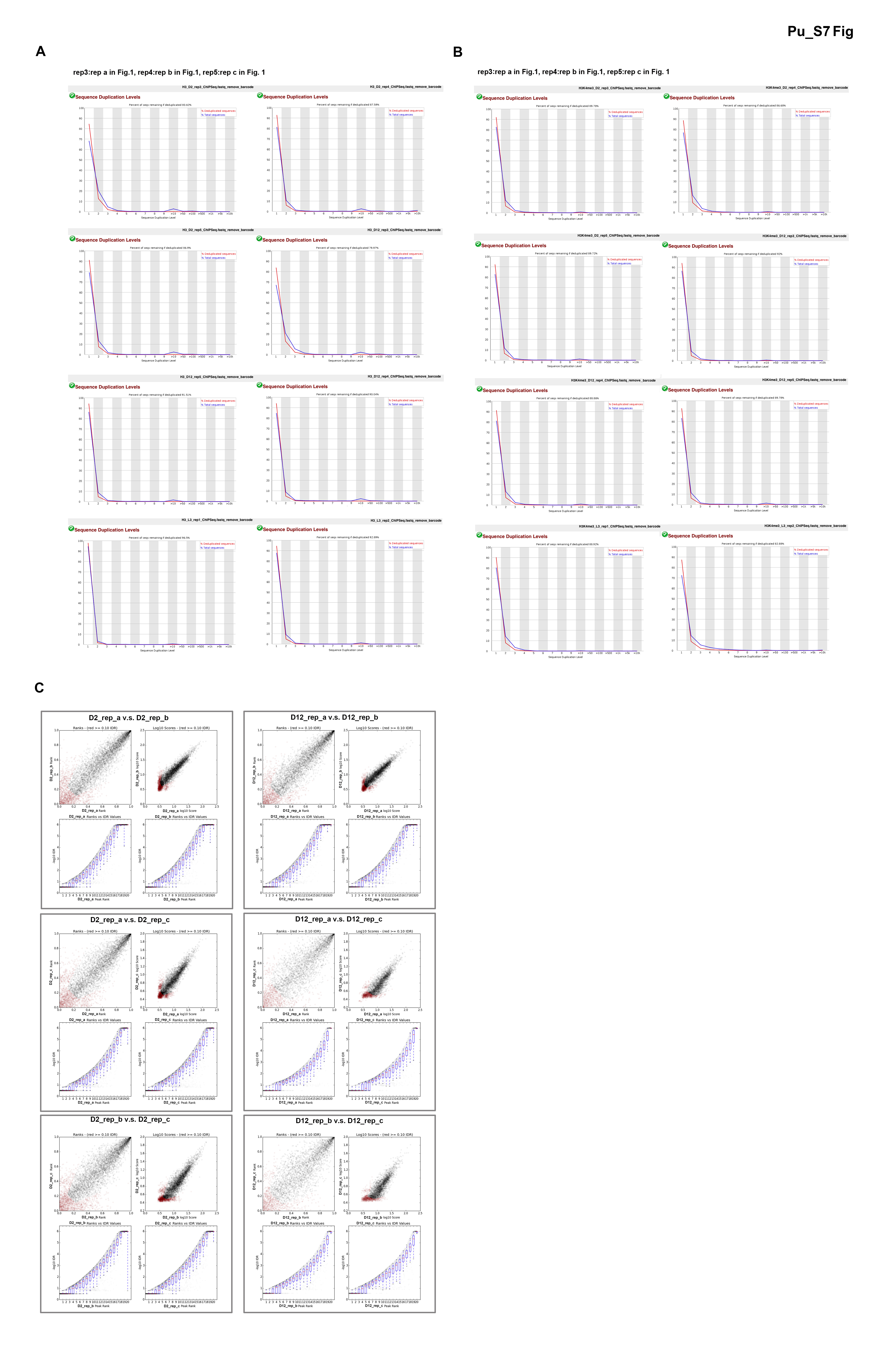

Supplement: S7 Fig — (A) Duplication plots for H3 ChIP-seq data. FASTQC module was used to analyze the duplication levels of each H3 ChIP-seq library and each plot shows the relative number of sequences with different degrees of duplication. First 100,000 sequences were analyzed for estimating the duplication levels in the whole data set. For each panel, the blue line is duplication distribution from all sequencing data, and the red one is from de-duplicated data. The proportion is the ratio of the deduplicated set relative to the original data. (B) Duplication plots for H3K4me3 ChIP-seq data. FASTQC module was used to analyze the duplication level of each H3K4me3 ChIP-seq library and each plot shows the relative number of sequences with different degrees of duplication. First 100,000 sequences were analyzed for estimating the duplication levels in the whole data set. For each panel, the blue line is duplication distribution from all sequencing data, and the red one is from de-duplicated data. The proportion is the ratio of the deduplicated set relative to the original data. (C)IDR analysis showing the consistency between replicates. H3K4me3 peaks were called by MACS2. Peaks from replicates at the same time point were compared and the results are shown for each pair. For each comparison: Upper Left: Replicates comparison based on peak ranks. The peaks that failed to pass the idr = 0.1 threshold are colored red. Upper right: Replicates comparison based on log10 peak scores. The peaks that failed to pass the idr = 0.1 threshold are colored red. Bottom left & right: Peaks rank versus idr scores are plotted from replicates. The boxplots display the distribution of idr values in each 5% quantile. (TIF) [file pgen.1007466.s007.tif]
