# Supplementary material for: Unique patterns of trimethylation of histone H3 lysine 4 are prone to changes during aging in Caenorhabditis elegans somatic cells
Source: PLoS Genet. 2018 Jun 18;14(6):e1007466. doi: 10.1371/journal.pgen.1007466 (PMC6023244; doi:10.1371/journal.pgen.1007466)
Supplement: S1 Text — (DOCX) [file pgen.1007466.s019.docx]

**Supplemental Methods**

**ChIP-seq data quality control**

Consistency of replicates

Consistency of replicates was measured by IDR (Irreproducible Discovery Rate) package in R using default settings, except --soft-idr-threshold was set at 0.1. (idr samples sample1 sample2 --soft-idr-threshold 0.1 --input-file-type narrowPeak --plot --output-file output)

ChIP-seq duplication measurement

Fastqc software was used for ChIPseq data duplication measurement. The raw sequencing data with the first 5-nt (homemade barcode) excluded were used.

Linear relationship among replicates

The H3K4me3 read counts were normalized by H3 read counts and library size. A 2kb sliding window size was used for calculating pair-wise Pearson correlation.
